# Intermittent Feedback-Control Strategy for Stabilizing Inverted Pendulum on Manually Controlled Cart as Analogy to Human Stick Balancing

**DOI:** 10.3389/fncom.2016.00034

**Published:** 2016-04-19

**Authors:** Naoya Yoshikawa, Yasuyuki Suzuki, Ken Kiyono, Taishin Nomura

**Affiliations:** Department of Mechanical Science and Bioengineering, Graduate School of Engineering Science, Osaka UniversityToyonaka, Japan

**Keywords:** intermittency, stable manifold, non-Gaussianity, posture control, stick balancing

## Abstract

The stabilization of an inverted pendulum on a manually controlled cart (cart-inverted-pendulum; CIP) in an upright position, which is analogous to balancing a stick on a fingertip, is considered in order to investigate how the human central nervous system (CNS) stabilizes unstable dynamics due to mechanical instability and time delays in neural feedback control. We explore the possibility that a type of intermittent time-delayed feedback control, which has been proposed for human postural control during quiet standing, is also a promising strategy for the CIP task and stick balancing on a fingertip. Such a strategy hypothesizes that the CNS exploits transient contracting dynamics along a stable manifold of a saddle-type unstable upright equilibrium of the inverted pendulum in the absence of control by inactivating neural feedback control intermittently for compensating delay-induced instability. To this end, the motions of a CIP stabilized by human subjects were experimentally acquired, and computational models of the system were employed to characterize the experimental behaviors. We first confirmed fat-tailed non-Gaussian temporal fluctuation in the acceleration distribution of the pendulum, as well as the power-law distributions of corrective cart movements for skilled subjects, which was previously reported for stick balancing. We then showed that the experimental behaviors could be better described by the models with an intermittent delayed feedback controller than by those with the conventional continuous delayed feedback controller, suggesting that the human CNS stabilizes the upright posture of the pendulum by utilizing the intermittent delayed feedback-control strategy.

## Introduction

Stick balancing, wherein experimental subjects are asked to stabilize a rigid stick on their fingertips in the vertically inverted position, is a typical motor task that has often been used for studying human motor control strategies exploited by the central nervous system (CNS) for stabilizing unstable dynamics. In such motor paradigms, the stabilization inevitably relies on sensory feedback information about the state of the controlled object, and the CNS must overcome multiple sources of instability, including feedback time delays (delay-induced instability), sensory uncertainty, endogenous motor noise, and the gravitational toppling torque inherent to the mechanical dynamics of the controlled object (Milton et al., 2008). For highly skilled subjects, the temporal fluctuation of the velocity increments (corresponding to the acceleration) of the stick are not Gaussian but exhibit a truncated Lévy distribution (Cabrera and Milton, 2004a; Cluff and Balasubramaniam, 2009). Moreover, corrective fingertip movements exhibit intermittent alternation between phases with extremely low movement amplitudes (off-phases) and those with high movement amplitudes (on-phases). This type of on-off intermittency can be characterized by the power-law distributions of the inter-corrective movement intervals (Cabrera and Milton, 2002).

Several types of neural control mechanisms underlying such motor behaviors during the stick-balancing or similarly during human quiet standing have been proposed. These include time-delayed feedback with multiplicative noise (Cabrera and Milton, 2002), model predictive controllers with a sensory uncertainty (Mehta and Schaal, 2002; Gawthrop et al., 2011; Loram et al., 2011; Insperger and Milton, 2014), act-and-wait control, whereby a delay-induced unstable system can be stabilized by the appropriate placement of a finite number of poles (eigenvalues), despite infinite dimensionality of the delay differential equations of the system, if the duration of periodically posed wait-phase with no active control is larger than the delay time (Insperger and Stepan, 2010), and time-delayed proportional-derivative-acceleration feedback control (Insperger and Milton, 2014). Here we consider another promising alternative: the intermittent time-delayed feedback controller (referred to as the intermittent feedback controller or the intermittent feedback-control strategy in this article) proposed for human posture control, whereby the mechanical dynamics of the human body during quiet standing are modeled as a controlled object by a single or a double inverted pendulum (Bottaro et al., 2008; Asai et al., 2009, 2013; Suzuki et al., 2012). The intermittent feedback-control model exploits the fact that the upright equilibrium posture with no active feedback control is characterized by a saddle-type instability accompanied by a hyperbolic vector field with stable and unstable manifolds in its phase space. Specifically, the action of the feedback controller is suspended intermittently (off-phase) when the state point of the inverted pendulum is close to the stable manifold, during which the transiently contracting dynamics (i.e., the motion of the state point approaching the unstable saddle-point) along the stable manifold are exploited for compensating the delay-induced instability. The feedback controller is then activated (on-phase) when the state point departs from the unstable equilibrium point, during which delay-induced unstable oscillatory dynamics bring the state point back to the stable manifold (*not to the equilibrium point*), triggering the onset of the off-phase. Alternation between the off- and on-phases (*both with unstable dynamics*) can lead to overall oscillatory and bounded stability in a robust manner (Bottaro et al., 2008; Asai et al., 2009; Suzuki et al., 2012; Asai et al., 2013). It has been argued that the slow dynamics near the saddle point with tiny additive motor noise are responsible for the power-law behaviors in the movement variability at a low frequency regime (Nomura et al., 2013).

The current article aims to demonstrate that a motor control strategy for stick balancing at the fingertip can also be characterized by intermittent feedback control. To this end, the stabilization of an inverted pendulum on a manually controlled cart (referred to as the cart-inverted-pendulum or CIP), which is analogous to the stick balancing on the fingertip, is considered to make the paradigm simpler and more tractable than balancing on the fingertip. The use of the CIP, rather than balancing on the fingertip, restricts the human motor actions to one-dimensional horizontal displacements of the pivot of the stick. Indeed, the stabilization of an inverted pendulum on the fingertip is achieved by three-dimensional (3D) movement of the fingertip; thus, control strategies other than the on-off intermittency of the feedback control can be employed, such as vertical periodic movements that can contribute to the stabilization through parametric resonance, as described by the Mathieu equation (e.g., Hoppensteadt, 2000). However, this sort of stabilization mechanism can be clearly ruled out as a strategy that the CNS might exploit for the task. Note that the movements of cart in the CIP task are confined to the frontal (medial-lateral) plane, whereas the movements tend to be in the sagittal (anterior-posterior) plane for stick balancing on the fingertip.

Our preliminary study demonstrated intermittent appearances of hyperbolic dynamics in the experimental CIP paradigm, reflecting the intermittent feedback-control strategy, via phase-space analysis and wavelet analysis of the experimental data (Yoshikawa et al., 2015). Here, we characterize the experimental behaviors of the CIP dynamics in detail, and the movement variability observed during CIP task is simulated by computational models of CIP control system with and without the intermittent time-delay feedback controller. We show that the CIP dynamics for skilled subjects can be better described by the models with the intermittent delay feedback controller than by those with the conventional continuous delay feedback controllers, suggesting that the human CNS stabilizes the upright posture of the pendulum by utilizing the intermittent feedback control strategy.

## Materials and methods

In this section, the computational models of CIP, the experimental procedure for the CIP task, and the time-series analysis methodology for characterizing the CIP movement variability are summarized.

### Computational models

#### CIP dynamics and stability of the inverted pendulum without feedback control

**Figure 1:**
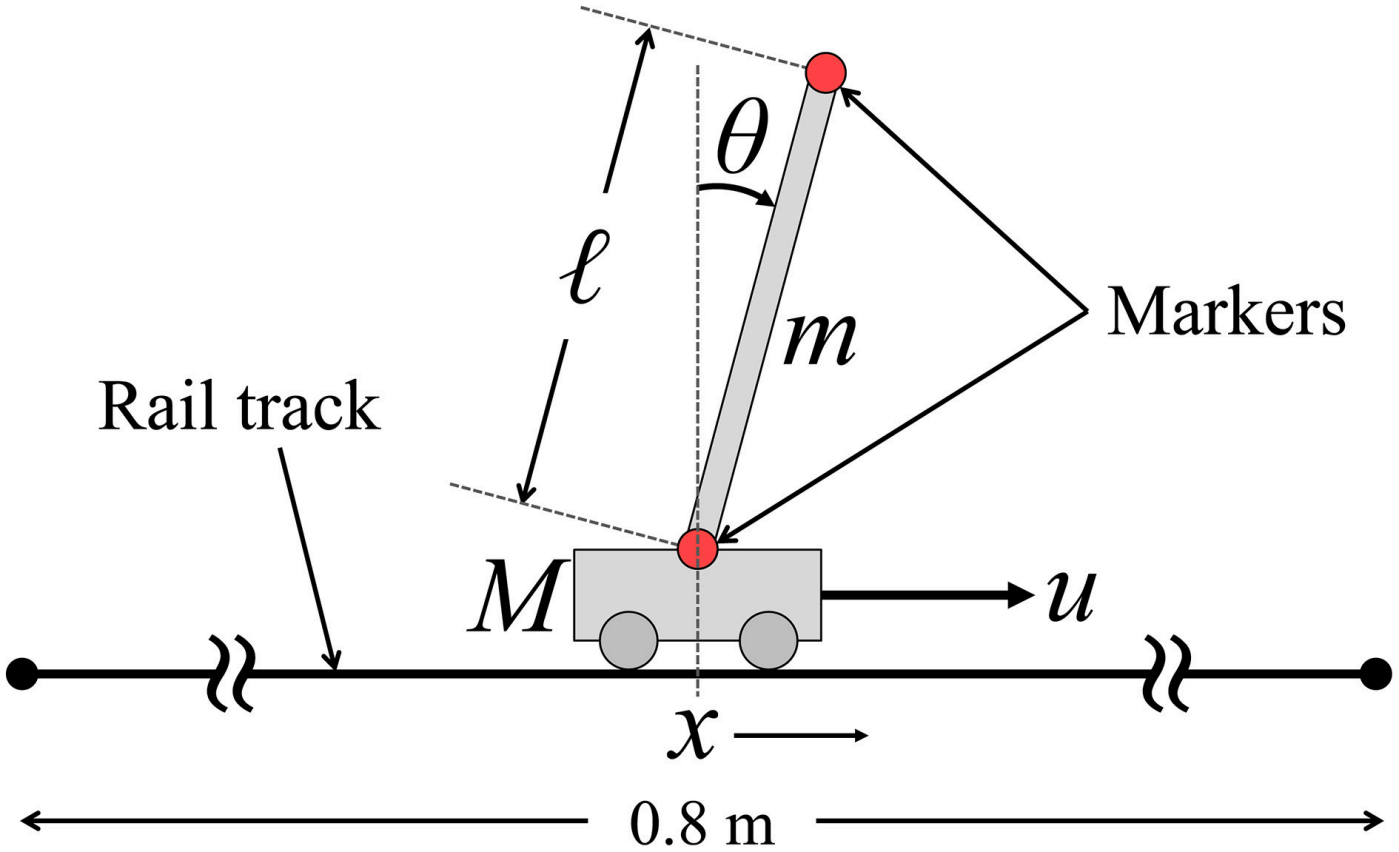
**The cart-inverted-pendulum (CIP) system**.

Figure 1 illustrates the CIP system that we consider in this study. The equations of motion of the CIP are described as follows:

(1) $$\frac{m}{3}\ell^{2}\ddot{\theta }+\frac{m\ell }{2}\ddot{x}=\frac{mg\ell }{2}\theta ,$$

(2) $$\frac{m\ell }{2}\ddot{\theta }+\left(M+m\right)\ddot{x}=u+\sigma \xi ,$$

where θ is the tilt-angle of the pendulum, *x* is the cart position from the origin along the rail, ℓ/2 is the distance from the joint to the center of mass of the pendulum, *mℓ*^2^/3 is the moment of inertia of the inverted pendulum around the joint, *m* is the pendulum mass, *M* (= 2*m*) is the cart mass, *g* is the gravitational acceleration, and *u* is the manual force exerted by the subject, which is a control input to the CIP system. See Milton et al. (2009) for detail. Moreover, we consider an additive force noise σξ, where ξ represents a Gaussian white noise with zero mean and unit variance and σ is the noise intensity (standard deviation of the noise). In our coordinate system, the positive (*x* > 0) and negative (*x* < 0) directions of the cart position correspond to the positive (θ > 0) and negative (θ < 0) directions of the tilt angle of the inverted pendulum. Equations (1) and (2) can then be rewritten in the state-space representation, as follows:

(3) $$\left[\begin{array}{c} \begin{array}{c} \dot{\theta }\\ \dot{\omega } \end{array}\\ \begin{array}{c} \dot{x}\\ \dot{v} \end{array} \end{array}\right]=[\begin{array}{cc} \begin{array}{cc} 0 & 1\\ A_{21} & 0 \end{array} & \begin{array}{cc} 0 & 0\\ 0 & 0 \end{array}\\ \begin{array}{cc} 0 & 0\\ A_{41} & 0 \end{array} & \begin{array}{cc} 0 & 1\\ 0 & 0 \end{array} \end{array}]\;\left[\begin{array}{c} \begin{array}{c} \theta \\ \omega \end{array}\\ \begin{array}{c} x\\ v \end{array} \end{array}\right]+\left[\begin{array}{c} \begin{array}{c} 0\\ B_{2} \end{array}\\ \begin{array}{c} 0\\ B_{4} \end{array} \end{array}\right](u+\sigma \xi ),$$

where $\omega =\overset{\cdot }{\theta }$ and *v* = ẋ are the angular velocity of the pendulum and the moving velocity of the cart, respectively. The elements of the system matrix and the input matrix are defined as:

$$A_{21}=\frac{2g}{\ell },\;A_{41}=-\frac{g}{3}$$

$$B_{2}=-\frac{2}{3m\ell }\;B_{4}=\frac{4}{9m}.$$

The origin (θ, ω, *x, v*) = 0 is an unstable fixed point (equilibrium point) of the mechanical system without control force (*u* = 0) and noise (σ = 0). The linear stability of this system with *u* = σ = 0 is determined by the solutions (eigenvalues) of the characteristic equation det(**A** − λ**E**) = 0, where **A** is the 4 × 4 matrix at the right-hand side of (3) and **E** is the 4 × 4 identity matrix, which can be derived as $\lambda_{\pm }=\pm \sqrt{2g/\ell }$ and λ_*d*_ = 0 (double-zero roots). The eigenvectors for λ_±_, denoted by **Φ**_±_, and the generalized eigenvectors for the double-zero eigenvalues, denoted by **Φ**_*d*1_ and **Φ**_*d*2_, are obtained as follows:

(4) $$\begin{array}{l} \Phi_{+}\;=\left[\begin{array}{c} \begin{array}{c} 1\\ \sqrt{2g/\ell } \end{array}\\ \begin{array}{c} -\ell /6\\ -\sqrt{g\ell /18} \end{array} \end{array}\right]\;,\Phi_{-}=\left[\begin{array}{c} \begin{array}{c} 1\\ -\sqrt{2g/\ell } \end{array}\\ \begin{array}{c} -\ell /6\\ \sqrt{g\ell /18} \end{array} \end{array}\right]\;,\Phi_{d1}=\left[\begin{array}{c} \begin{array}{c} 0\\ 0 \end{array}\\ \begin{array}{c} 1\\ 0 \end{array} \end{array}\right]\;,\\ \Phi_{d2}=\left[\begin{array}{c} \begin{array}{c} 0\\ 0 \end{array}\\ \begin{array}{c} 0\\ 1 \end{array} \end{array}\right] \end{array}$$

We chose two unit vectors parallel to the *x* and *v* axes as the generalized eigenvectors corresponding to double-zero eigenvalues λ_*d*_, which are independent of the stability of the pendulum in the θ-ω plane (thus, detailed descriptions of λ_*d*_ are omitted in this paper). Because the other two eigenvalues λ_±_ are real numbers with opposite signs, the system has a one-dimensional stable manifold and a one-dimensional unstable manifold in the phase space without a control force. In particular, the stable and the unstable manifolds are represented as lines that pass through the origin in the θ-ω plane, and they are parallel to the vectors $\Phi_{-}^{\prime }={\left[1-\sqrt{2g/\ell }\right]}^{\text{T}}$ and $\Phi_{+}^{\prime }={\left[1\sqrt{2g/\ell }\right]}^{\text{T}}$, respectively.

The dynamics of θ and ω are independent of *x* and *v*, because the (1, 3), (1, 4), (2, 3), and (2, 4) elements of matrix **A** are all zeros. That is, as far as we consider the θ-ω plane in the phase space, the motion of the point (θ, ω) is independent of the cart position and its velocity if *u* = 0. (Note that the dynamics of θ and ω are influenced by the cart dynamics if *u* ≠ 0 and it depends on *x* and/or *v*). In this way, the upright position (θ, ω) = (0, 0) of the pendulum can be considered as a saddle-type unstable equilibrium point when no control force is applied to the cart. Because the upright equilibrium point of a standard inverted pendulum model with a uniaxial joint fixed in the space is also saddle type (Asai et al., 2009), the inverted pendulum for a CIP without control force behaves similarly to the standard inverted pendulum in the θ-ω plane. This suggests that the experimental subjects can exploit transiently contracting dynamics along the stable manifold of the non-controlled inverted pendulum as in the intermittent control model of posture control for human quiet standing (Asai et al., 2009, 2013).

#### Time-delayed feedback controller and the continuous feedback-control model

In this study, the manual control force *u* is modeled by the following time-delayed proportional and derivative feedback controller

(5) $$u(t)=P_{\theta }\theta_{△}+D_{\theta }\omega_{△}+D_{x}v_{△},$$

where θ_Δ_ = θ(*t* − Δ), ω_Δ_ = ω(*t* − Δ), and *v*_Δ_ = *v*(*t* − Δ) are the delayed state variables with a feedback delay time Δ; *P*_θ_, and *D*_θ_ are the proportional and derivative feedback gains for the angular and angular-velocity deviations from the upright equilibrium point, respectively; and *D*_*x*_ is the derivative feedback gain for the deviation of the cart position from the null velocity. Because we did not ask the subjects to keep the cart at a specific position on the rail, a feedback force proportional to the cart position was not included in the modeling of the manual control force *u*. Our preliminary examinations on the model showed that the use of the proportional feedback controller with respect to the cart position diminishes the stability region of the CIP system substantially, and we plan to investigate this in the future.

**Figure 2:**
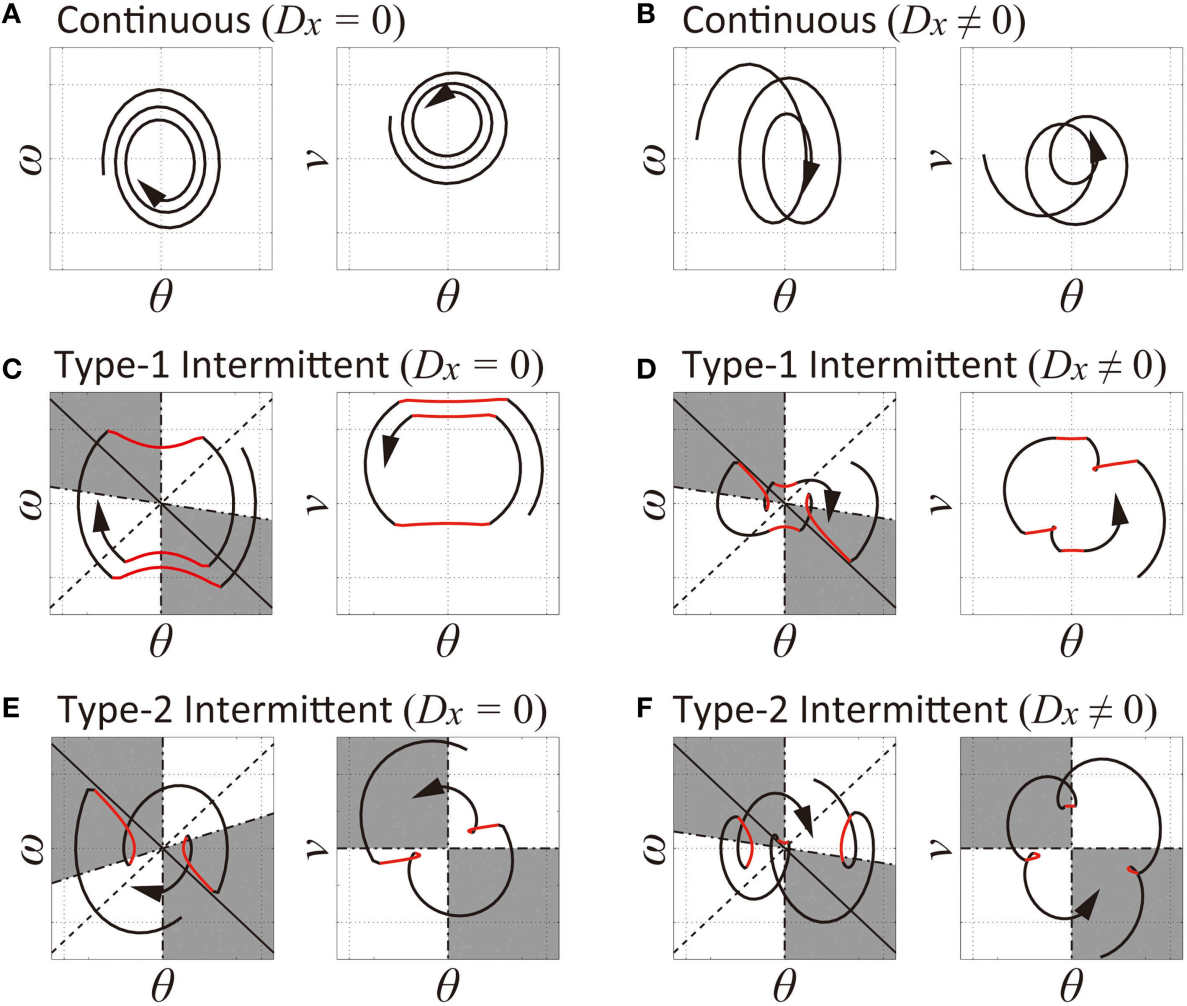
**Off- and on-regions in the θ-ω plane and the θ-*v* plane to define the switching conditions between the off-phase and on-phase of the feedback control**. In each plane, the white and gray regions represent the on-region and the off-region, respectively. **(A,B)** Continuous control model with *D*_*x*_ = 0 and *D*_*x*_ > 0, respectively: the on-region occupies the whole of the θ-ω and the θ-*v* planes for both cases. **(C,D)** Type-1 intermittent control model with *D*_*x*_ = 0 and *D*_*x*_ > 0, respectively: the off-regions in the θ-ω plane are located near the stable manifold (depicted as a solid line with a negative slope passing though the origin) for the inverted pendulum with no feedback control. The θ-*v* plane does not have off-regions. **(E,F)** Type-2 intermittent control model with *D*_*x*_ = 0 and *D*_*x*_ > 0, respectively: onset of the off-phase is determined based on the off-regions in the θ-ω phase and those in the θ-*v* plane. For each panel, the thick curve with arrowheads represents a sample trajectory of the model, and the black and red curves represent the trajectories during on-phases and off-phases, respectively. The circular shape of the black trajectories is due to the delay-induced instability with delay feedback control, and the hyperbolic shape of the red trajectories move along the stable manifold and/or the unstable manifold (depicted as a dotted line with a positive slope) during off-phases. See text for details.

A simple and conventional model of the manual CIP system, referred to as the continuous (delayed) feedback-control model (or continuous control model), is used as a reference against models with intermittent controllers. In the continuous control model, the manual force *u* defined by Equation (5) is always applied to the cart, independent of the cart position and the posture of the inverted pendulum (Figure 2). Although, the origin (θ, ω, *x, v*) = 0 is a fixed point of the continuous control model, we are not necessarily interested in its stability, since the cart position may vary along the rail track while the pendulum is stabilized by the subject. Instead, we analyze behaviors of the pendulum using a pseudo-equilibrium point $\left(\bar{\theta },\bar{\omega }\right)$ of the system in the θ-ω plane, which is defined as a solution of the zeros in the right-hand-side of the first and second rows of Equation (3). Namely,

(6) $$\dot{\theta }=\omega =0,$$

(7) $$\dot{\omega }=\frac{2g}{\ell }\theta -\frac{2}{3m\ell }\left(P_{\theta }\theta_{△}+D_{\theta }\omega_{△}+D_{x}v_{△}\right)=0.$$

From Equation (6), we have $\bar{\omega }=0$, and thus ω_Δ_ = 0 and $\theta =\theta_{\Delta }=\bar{\theta }$, which yields the pseudo-equilibrium point using Equation (7) as follows:

(8) $$\left(\bar{\theta },\bar{\omega }\right)=\left(\begin{array}{cc} \frac{-D_{x}v_{△}}{P_{\theta }-3mg}, & 0 \end{array}\right).$$

The pseudo-equilibrium point is not a fixed point, but it moves right and left as the function of the delayed cart velocity *v*_Δ_. If *P*_θ_ > 3*mg* as in our experimental setup, $\bar{\theta }<0$ if *v*_Δ_ > 0, and $\bar{\theta }>0$ if *v*_Δ_ < 0. In other words, the moving pseudo-equilibrium point is located on the θ-axis of the left and right halves in the θ-ω plane, respectively, if the cart moves rightward and leftward Δ (s) in the past. Thus, a state point in the θ-ω plane may pursue the pseudo-equilibrium point located on the negative and positive parts of the θ-axis, respectively, for *v*_Δ_ > 0 and *v*_Δ_ < 0. By approximating $\theta_{\Delta }\approx \theta -\Delta \overset{{}^{\circ}}{\theta }$ and $\omega_{\Delta }\approx \omega -\Delta \overset{{}^{\circ}}{\omega }$, and by defining $\theta \approx \bar{\theta }+\vartheta$, Equations (6, 7) can be rewritten as:

(9) $$\begin{array}{l} \left(3m\ell -2\Delta D_{\theta }\right)\ddot{\vartheta }+2\left(D_{\theta }-△P_{\theta }\right)\dot{\vartheta }\\ \;+\;2\left(P_{\theta }-3mg\right)\vartheta =0. \end{array}$$

Note that, although Taylor series expansion of delayed terms with respect to the delay might give a good approximation of the dynamics is some cases, such approximation has no mathematical foundations. Interestingly, first-order expansion may approximate stability properties, but higher order expansions results in an unstable system [see Insperger (2015) for detailed discussion about this]. The pseudo-equilibrium point is stable if and only if:

(10) $$\frac{D_{\theta }-△P_{\theta }}{3m\ell -2\Delta D_{\theta }}>0\text{ and }\frac{P_{\theta }-3mg}{3m\ell -2\Delta D_{\theta }}>0.$$

Moreover, the pseudo-equilibrium point is topologically focus-type if:

(11) $${\left(\frac{D_{\theta }-△P_{\theta }}{3m\ell -2\Delta D_{\theta }}\right)}^{2}-\frac{2\left(P_{\theta }-3mg\right)}{3m\ell -2\Delta D_{\theta }}<0.$$

For example, for a large *P*_θ_ with small *D*_θ_ that do not fulfill Equation (10) but fulfill Equation (11), dynamics of the pendulum in the θ-ω plane may be approximated by a growing oscillation, circulating clock-wise around the pseudo-equilibrium points that are moving at the left or the right sides of the origin, depending on the sign of *v*_Δ_. If the cart moves from side to side for pursuing and catching-up the falling pendulum, the sign of *v*_Δ_ may alternate, leading to an alternation between oscillations around the pseudo-equilibrium points at the left and the right sides of the origin (Figure 2B). Note that the origin is the pseudo-equilibrium point if *D*_*x*_ = 0, for which the state point draws a simple circular trajectory in the θ-ω plane (Figure 2A).

A linear stability analysis of the delay differential equation (the continuous control model)

(12) $$\ddot{\theta }=\frac{2g}{\ell }\theta -\frac{2}{3m\ell }P_{\theta }\theta_{\Delta }-\frac{2}{3m\ell }D_{\theta }{\dot{\theta }}_{\Delta },$$

which corresponds to the second row of Equation (3) when using Equation (5) with *D*_*x*_ = 0, revealed the following critical conditions for the existence of a stability region in the *P*_θ_-*D*_θ_ parameter plane (Stepan, 2009). For a pendulum length ℓ, the critical delay time Δ_*cr*_ can be derived as:

(13) $${△}_{cr}=\sqrt{\frac{\ell }{g}}.$$

That is, for a given ℓ, there is no stability region in the *P*_θ_-*D*_θ_ parameter plane for any delay time Δ > Δ_*cr*_. Similarly, for a given delay time Δ, the critical length of the pendulum ℓ_*cr*_ can be derived as:

(14) $$\ell_{cr}=g\Delta^{2}.$$

That is, for a given delay time Δ, there is no stability region in the *P*_θ_-*D*_θ_ parameter plane for any pendulum length ℓ < ℓ_*cr*_. For our experimental CIP system, the critical delay is calculated as Δ_*cr*_ = 0.226 s with ℓ = 0.5 m. The critical length is ℓ_*cr*_ = 0.098 m if we assume Δ = 0.1 s and ℓ_*cr*_ = 0.392 m if we assume Δ = 0.2 s. Because the feedback delay time is estimated to be between 0.08 and 0.22 s (Mehta and Schaal, 2002; Cabrera and Milton, 2004a), the length of the pendulum (ℓ = 0.5 m) used in this study is slightly greater than but very close to the critical length. Thus, theoretically, the parameters of the CIP system used in this study are set scarcely within the range of stability region, and the subjects can stabilize the inverted pendulum even if they utilize the continuous time-delayed proportional and derivative feedback control for the tilt angle and the angular velocity of the pendulum with appropriately tuned gains. With this condition, we are interested in whether the experimental behaviors of the subjects can be well described by the continuous control model or the intermittent control model.

#### Type-1 intermittent feedback-control model

We consider two types of intermittent feedback-control models. Both models assume the time-delayed feedback controller defined by Equation (5), but the conditions for the state-dependent inactivation and activation of the feedback controller differ slightly. In one type of model, referred to as the type-1 intermittent control model, the conditions for switching between the off-phase without the feedback control force and the on-phase with the feedback control force depend only on the delayed state of the pendulum, and the intermittent manual feedback control is defined as follows:

(15) $$u=\{\begin{array}{cc} 0, & \text{if }\theta_{△}\left(\omega_{△}-a\theta_{△}\right)<0,\\ P_{\theta }\theta_{△}+D_{\theta }\omega_{△}+D_{x}v_{△}, & \text{otherwise}, \end{array}$$

where the parameter *a* takes a value within [−∞, +∞] that determines the slope of the switching boundary in the θ-ω plane (Figures 2C,D). This state-dependent switching rule is exactly the same as the one proposed for human posture control during quiet standing (Asai et al., 2009). According to Figure 2C for *D*_*x*_ = 0 and Figure 2D for *D*_*x*_ > 0, the feedback controller is turned off (inactivated) if the delayed state of the pendulum (θ_Δ_, ω_Δ_) is located in the gray colored area (off-region) in the θ-ω plane, in the middle of which the stable manifold settles, and otherwise (on-region), it is turned on (activated). In this way, the state point can receive benefit of the stable manifold along which the state point approaches the saddle-point transiently. As in the case of Figure 2C, a trajectory for the state of the pendulum in the θ-ω plane exhibits hyperbolic curves (the red curves in Figure 2C) according to the hyperbolic vector field around the saddle point at the origin as far as the delayed state (θ_Δ_, ω_Δ_) is located in the off-region, and it forms *peanut-shaped* cycles together with the arc-like segments for the periods of on-phases. The corresponding trajectory in the θ-*v* plane when the feedback controller is switched-off forms almost horizontal segment, implying null-acceleration, because no control force is applied to the cart during the off-phases. A trajectory of the model for *D*_*x*_ > 0 (Figure 2D) behaves similarly. However, because of the existence of the *v*_Δ_-dependent pseudo-equilibrium point, the state point moves around the pseudo-equilibrium points that appear at the left and the right sides of the origin depending on the sign of *v*_Δ_ (Figure 2D) during the on-phases. Because the sign of *v*_Δ_ typically alternates when the pendulum swings to right and left, the pseudo-equilibrium point follows it, and thus the trajectory tends to be elongated horizontally, leading to a trajectory more like *butterfly-wings* rather than the peanut.

The off-region in the θ-ω plane disappears in the limit of *a* → −∞, whereby the type-1 intermittent control model becomes identical to the continuous control model. On the other hand, the whole θ-ω plane becomes off-region in the limit of *a* → +∞, for which the upright position of the pendulum can never be stabilized. Thus, the type-1 intermittent control model and the continuous control model are described by identical system equations that are parameterized by a single parameter a, meaning that one can analyze how the dynamics of the model change as the controller transits from continuous to intermittent feedback.

#### Type-2 intermittent feedback-control model

In the other type of intermittent control model, referred to as the type-2 intermittent control model, the conditions for switching between the off-phase and the on-phase depend on the delayed state of the pendulum as well as the cart velocity (Figures 2E,F), and the corresponding manual feedback control is defined as follows:

(16) $$u=\{\begin{array}{cc} 0, & \begin{array}{c} \text{if }\theta_{△}\left(\omega_{△}-a\theta_{△}\right)<0\\ \text{and }\theta_{△}v_{△}<0\text{ and }u_{△}\neq 0, \end{array}\\ & \text{or if }\theta_{△}\omega_{△}<0\text{ and }u_{△}=0,\;\\ P_{\theta }\theta_{△}+D_{\theta }\omega_{△} & \text{otherwise}\\ \;+\;D_{x}v_{△}, & \end{array}$$

where *u*_Δ_ = *u*(*t* − Δ). Roughly speaking, by the switching condition of Equation (16), the active feedback control is turned off when the state point is close to the stable manifold as in Equation (15) and the cart velocity is close to zero. Because we frequently encountered chattering-like behaviors for this model during our preliminary model simulations, the dead-time (Δs) was introduced for the type-2 intermittent control model, whereby switching between the off-phase and on-phase was prohibited within this dead-time. The switching condition θ_Δ_*v*_Δ_ < 0 for triggering the off-phase and *u*_Δ_-dependent condition θ_Δ_ω_Δ_ < 0 for terminating the off-phase were introduced for the type-2 intermittent control model, based on our preliminary analysis of the experimental behaviors, where we observed that the amplitude of the manual control force estimated by the cart acceleration tended to decrease (presumably corresponding to the onset of off-phase) with respect to the pendulum tilt angle. That is, such estimated onsets of the off-phase tended to occur either when the cart moved with a positive velocity and approached zero with a negative tilt angle or, oppositely, when the cart moved with a negative velocity and approached zero with a positive tilt angle, i.e., immediately after the cart overtook the top (or the center of mass) of the pendulum, where the cart acceleration is locally maximized in both cases (Yoshikawa et al., 2015). The effects of the additional switching conditions on the CIP dynamics are not trivial; on one hand, the additional conditions for triggering the off-phase may increase the duration of the on-phase with feedback control, but on the other hand, derivative feedback control for the cart velocity may prevent the cart from gaining a large velocity, leading to the suppression of the generation of too much feedback control force.

The switching conditions in the for the type-2 intermittent control provides an alternative mechanism that can produce a butterfly-wings-like trajectory in the θ-ω plane, as in the type-1 intermittent control model with the pseudo-equilibrium point (Figure 2D), even without the pseudo-equilibrium point for *D*_*x*_ = 0 by using a combination of the off-regions in the θ-ω plane and those in the θ-*v* plane (Figure 2E). In Figure 2E, for example, the state point draws a hyperbolic curve (red-colored) in the left half plane along the stable manifold during the off-phase, which is initiated by the coincidence of two events: entrance of the state point in the off-regions both in the θ-ω and the θ-*v* planes and terminated almost immediately after the red curve tumbles out of the second quadrant of the θ-ω plane, leading to the reversal of the trajectory moving upward and then to the right half plane with the feedback control force (the black arc that is terminated at the off-region in the right half plane, just before the state point reaches the stable manifold). When *D*_*x*_ > 0, the system has the pseudo-equilibrium point during the on-phase, and thus, the butterfly-wings-like trajectory is generated by the combination of this switching mechanism and the right-left alternation of the pseudo-equilibrium point (Figure 2F).

In this way, both of the type-1 and the type-2 intermittent control model exhibit the butterfly-wings-like trajectory that is commonly characterized by the changes in the sign of ω both in the right and left half plane in the θ-ω plane. A behavioral difference between the type-1 and the type-2 models is that the state point passes through the upright position (θ = 0, i.e., the motion between the right-half and the left-half of the θ-ω plane) is achieved typically during the off-phase in the type-1 model, whereas it is achieved typically during the on-phase in the type-2 model. In other words, the type-1 controller catches the freely falling pendulum, and then just throws (or kicks) the pendulum up impulsively so that the pendulum can approach the upright position freely without the control force, whereas the type-2 controller also catches the freely falling pendulum, but the pendulum is brought to the other side actively by the control force.

As in the type-1 intermittent control model, the off-region in the θ-ω plane disappears in the limit of *a* → −∞, whereby the type-2 intermittent control model also becomes identical to the continuous control model, as the two conditions for the off-phase described in Equation (16) can never be satisfied.

#### Model simulations

The dynamics of the continuous and two types of intermittent control models were numerically simulated using the forward Euler method for stochastic differential equations with a time step of 0.001 s (Kloeden and Platen, 1992). The feedback delay time was fixed at Δ = 0.1 s throughout the study. For a given model with a set of parameter values (see Section Stability Analysis of the Models and Fitting Experimental Behaviors to the Models for details regarding the parameters), the model was integrated for a time span of 70 s with 100 different initial conditions, where (θ(0), ω(0)) were randomly selected from the uniform distribution between [−0.05, 0.05] for the initial tilt angle and the angular velocity, and (*x*(0), *v*(0)) were set as zero at time *t* = 0. The initial values (the initial functions) for *t* ∈ [−Δ, 0) were all set as zero for all the 100 initial conditions.

The dynamics of a model with a given set of the parameters were determined as stable if the pendulum of the model did not fall (i.e., *max*|θ(*t*)| < π/2) for all 100 simulated trials with 100 different initial conditions. The threshold value π/2 for falling condition might be too large, but we used this value to avoid failures in detecting events of “big risk of falls” where the falling pendulum is recovered from a very large tilt angle. For each of the stable solutions, transient data for the first 10 s were discarded to obtain 100 sets of the steady-state solution 60 s in length (the same length as the segmented experimental skilled data). Then, the data were down-sampled at 60 Hz and smoothed by a low-pass filter with a cutoff frequency of 10 Hz.

### Experimental methods

In the manual stabilization of the CIP, an inverted pendulum is attached on a cart by a uniaxial free joint, and the control objective is to stabilize the upright posture of the pendulum by moving the cart translationally, based on the visual (and tactile) feedback information regarding the posture of the pendulum. The rotational motion of the inverted pendulum is not controlled by a direct joint torque but is controlled indirectly through the acceleration of the cart, which is manually repositioned in a horizontal direction by the hand of the experimental subject. A relatively large sensory feedback delay time is indispensable owing to neural transmission and neural information processing performed by the CNS of the subject. In the CIP without a feedback time delay, the upright posture of the inverted pendulum can easily be stabilized asymptotically by using, for example, a simple proportional-derivative control with appropriate (optimal) feedback gains. However, delay-induced instability may occur in the case of feedback control with delay (Insperger and Milton, 2014), where the neural transmission delay has been estimated as 0.08–0.22 s in stick balancing on the fingertip (Mehta and Schaal, 2002; Cabrera and Milton, 2004a). The delay time is sufficiently large to cause the delay-induced instability. Nevertheless, skilled subjects can stabilize the stick in the upright position in a robust manner.

#### Experimental CIP task

The CIP as in Figure 1 was assembled mechanically, and the manual-control performances were measured for 12 healthy young subjects (aged 21–28 years, all right-handed). All the subjects provided written informed consent to participate in this research, which was approved by the ethical committee for human studies at the Graduate School of Engineering Science, Osaka University. The CIP comprised a uniform-density thin, rigid stick (pendulum) with a length of ℓ = 0.5 m and a mass of *m* = 0.125 kg, as well as a cart with a mass of *M* = 2*m* = 0.25 kg. The cart could slide smoothly along a rail track 0.8 m in length. The cart and the stick were joined by a uniaxial bearing; thus, the joint was considered as a frictionless pin joint. Therefore, the pendulum could rotate only in the two-dimensional plane along the rail track. The subjects manipulated the cart on the track by holding a handle attached to the cart with their right hand. 3D optical motion capture with five infrared cameras (BTS, Milan, Italy) was performed to measure the motion of the infrared reflection markers attached to both ends of the stick during the task with a sampling frequency of 300 Hz.

Each subject performed the task for 4–7 days, at most 10 trials per a day. They practiced the task for 2 min prior to the first measured trial each day. The balancing-time duration, *d* (s), was defined as the time interval from the instant when the subject released the stick supported by the left hand to the instant when the stick fell down. For each measurement day, the total balancing-time duration, i.e., the sum of the balancing times *d*, was acquired, and the trials were terminated when the total duration exceeded 600 s. Performances with *d* > 70 s among all trials throughout the measurement days were considered as skilled performances. For each subject, the experiment was terminated either when the total number of skilled performances exceeded 10 or when the total number of measurement days reached seven. Subjects exhibiting more than 10 skilled performances were considered as skilled performers. In this study, the movement variability during the CIP task was analyzed only for the skilled performers.

#### Data preparation for skilled performances

For each skilled subject, the skilled performances were ranked based on the balancing-time duration, from longest to shortest. Then, the longest duration *d*_*max*_ (s) was defined for each subject in order to rank the subjects based on their skill level for the task. For the skilled-performance data, the transient behaviors for the first 10 s of the trial were discarded. The remaining steady state-data were divided into segments with a duration of 60 s (where the last segment less than 60 s was also discarded), and the segments were analyzed separately to characterize the skilled performances. The steady-state durations were split into 60 s windows in order to perform the ensemble average of time series statistics under identical conditions across subjects. Specifically, for each subject, 10 segments were taken, from the longest data first and then from the second- and third-longest data, and each segment was analyzed separately. We also defined the mean duration *d*_*mean*_ (s) for each subject, which is the mean value of the balancing-time durations for the skilled performances that were used for collecting the 10 segments. A longer balancing duration of the best, second-best, and third-best trials led to a smaller number of trials necessary for collecting the 10 data segments.

For data analysis, time-series data of the *x*-coordinate values for each of two markers attached to both ends of the stick were analyzed after they were down-sampled at 60 Hz, where the *x*-axis was defined along the rail. The time series of the *x*-coordinate position of the markers at the bottom and top of the stick were denoted as *m*_*b*_[*n*] and *m*_*t*_[*n*], respectively, where *n* is the discretized time step. We defined *m*_*b*_[*n*] and *m*_*t*_[*n*] so that the origin was located at the bottom end of the stick, as follows:

$${\tilde{m}}_{b}\left[n\right]=m_{b}\left[n\right]-{\bar{m}}_{b},{\tilde{m}}_{t}\left[n\right]=m_{t}\left[n\right]-{\bar{m}}_{b},$$

where ${\bar{m}}_{b}$ is the average of *m*_*b*_[*n*]. The tilt-angle θ of the pendulum, its angular velocity ω, and the cart position *x*, velocity *v*, and acceleration α were then calculated using *m*_*b*_[*n*] and *m*_*t*_[*n*], respectively, as follows:

$$\theta \left[n\right]=\sin^{-1}\frac{{\tilde{m}}_{t}\left[n\right]-{\tilde{m}}_{b}\left[n\right]}{\ell }\;,\;\omega \left[n\right]=\frac{\theta \left[n+1\right]-\theta \left[n-1\right]}{2\Delta t}$$

and

$$\begin{array}{l} x\left[n\right]={\tilde{m}}_{b}\left[n\right],\;v\left[n\right]=\frac{x\left[n+1\right]-x\left[n-1\right]}{2\Delta t},\\ \;\alpha \left[\text{n}\right]=\frac{x\left[n+2\right]-2x\left[n\right]+x[n-2]}{{\left(2\Delta t\right)}^{2}}, \end{array}$$

where Δ*t* = 1/60 s is the resampling time step. The resulting data were low-pass filtered offline by using a fourth-order zero phase-lag Butterworth filter with a cutoff frequency of 10 Hz.

### Characterizing movement variability

The movement variability during the experimental CIP task and that in the computational models were quantified using five summary measures (indices): two indices for the non-Gaussianity of the cart-acceleration distributions, the distribution of the time intervals of corrective movements, the characteristic peak frequency in the power spectra for the motion of the pendulum, and the standard deviation of the motion of the pendulum, as summarized in this subsection. Those five indices were used not only for characterizing the experimental and simulated movement variability, but also to estimate the parameter values of the models compared to experimental data. These indices were calculated for each 60-s time series. For the experimental data, the movement variability of the pendulum for each subject was characterized by the mean value of each of the five indices averaged over the ten-segmented data. Similarly, for the simulated data in the models, the movement variability of the pendulum for the model with every examined set of the parameters was characterized by the mean value of each of the five indices averaged over the 100 initial conditions.

#### Non-gaussianity measures for the cart-acceleration distributions

The movement variability during stick balancing at the fingertip exhibits non-Gaussian fluctuations (Cabrera and Milton, 2002). To characterize such fluctuations, we employed a multiplicative lognormal distribution in which the multiplication of Gaussian and log normally distributed random variables are assumed. This distribution was originally introduced as a model for describing velocity fluctuations in fully developed turbulent flows (Castaing et al., 1990). Subsequently, it was shown that this distribution provides a good approximation of non-Gaussian distributions observed in a variety of systems (Kiyono et al., 2004, 2006; Manshour et al., 2009).

A multiplicative lognormal distribution of a random variable *X* with zero mean and unit variance is given by the following equation^1^:

(17) $$\begin{array}{l} P_{\lambda }\left(x\right)=\int_{0}^{\infty }\frac{1}{\sqrt{2\pi }\lambda }\exp\left(-\frac{{\left(\ln\sigma \;+\lambda^{2}\right)}^{2}}{2\lambda^{2}}\right)\;\\ \;\frac{1}{\sqrt{2\pi }\sigma }\exp\left(-\frac{x^{2}}{2\sigma^{2}}\right)d(\ln\sigma \;)\;, \end{array}$$

where *x* is the outcome of the random variable *X*, and σ is the integration variable describing the fluctuating standard deviation of the Gaussian distribution. The scalar index λ is a shape parameter quantifying the non-Gaussianity of the distribution *P*_λ_(*x*). By taking the limit λ?0 in Equation (17), a Gaussian distribution is obtained. On the other hand, a larger l indicates a non-Gaussian distribution with fatter tails and a more peaked center (a large Kurtosis) compared with a Gaussian distribution. To estimate the non-Gaussianity parameter λ^2^ from the observed data {*x*_*i*_}, the following moment-based estimator was proposed (Kiyono et al., 2007):

(18) $$\lambda_{q}^{2}=\frac{2}{q\left(q-2\right)}\left[\ln\left(\frac{\sqrt{\pi }\text{ E}[{\left|x\right|}^{q}]}{2^{q/2}}\right)-\ln\;\Gamma \left(\frac{q+1}{2}\right)\;\right],$$

where E is the expectation function, *q* is the order of the moment with *q* ≠ 0, 2, and Γ is the gamma function. By taking *q* → 0, we obtain:

(19) $$\lambda_{q\to 0}^{2}=-\text{ E}\left[\ln\left|x\right|\;\right]-\frac{\gamma +\ln2\;}{2},$$

where γ ≈ 0.57721566 is the Euler-Mascheroni constant. If *x* obeys a multiplicative lognormal distribution, and the sample number of *x* is infinity, the estimated value of λ^2^ is a constant independent of *q*. However, in a practical situation with finite samples, the estimated value of λ^2^ depends on *q*. In this paper, to reduce the effect of very large outliers, we estimate the value of λ^2^ by using $\lambda_{q\to 0}^{2}$ and $\lambda_{q=1}^{2}$. The estimate using $\lambda_{q\to 0}^{2}$ emphasizes samples located in the center part of the distribution, and that using $\lambda_{q=1}^{2}$ emphasizes samples located slightly outside of the distribution tails. A large difference between $\lambda_{q\to 0}^{2}$ and $\lambda_{q=1}^{2}$ indicates a large deviation from the multiplicative lognormal distribution. Using these estimates, we characterized the distributions of the measured acceleration data.

#### Distributions of inter-corrective movement intervals (ICM distributions)

Temporal series of the tilt angle θ of the pendulum during (experimental and simulated) balancing were analyzed in order to detect a sequence of active interventions, i.e., corrective movements. As in human stick balancing (Cabrera and Milton, 2002), fluctuations in the tilt angle exhibit the following characteristics: (i) periods in which small fluctuations occur alternate with shorter periods characterized by larger changes; (ii) the baseline for the fluctuations is not the upright position; i.e., most of the time, the balanced stick deviates slightly from the vertical. We analyzed the changes in a time series of cosθ, which is close to unity during periods with small fluctuation amplitudes. We consider that time instants when the value of cosθ falls below a given threshold that is close to unity correspond to onsets of the corrective movements. Accordingly, a sequence of inter-corrective movement (ICM) intervals was obtained. A time average value of cosθ over each balancing trial, which should be close to unity for small variations of θ during successful performance, was defined as the threshold value. ICM distributions were characterized by the median of the distribution (ICM-median) and the slope of the linear fitting of the distribution (ICM-slope). The linear fitting for the ICM-slope was performed for the range of ICMs above the ICM-median. Because our preliminary examinations revealed that the ICM-slope was sensitive to the threshold value for detecting the corrective movements, we decided not to use this index for characterizing dynamics of the experimental and model-simulated data, but just for reference.

#### Characteristic peak frequency in the power spectra for the motion of the pendulum

Power spectral density (PSD) functions of the tilt-angle fluctuations were estimated by taking a fast Fourier transform of each segmented time-series spanning 60 s. Then, the ensemble average of those PSDs was considered as the PSD of the tilt-angle fluctuation for each subject and for each model with a given set of parameter values. Preliminary PSD analysis showed that the PSD always exhibited a characteristic peak. Thus, the PSD of each subject was characterized by the peak frequency (PSD-PF).

#### Standard deviation of motion of the pendulum

The size of fluctuations of the tilt-angle of the pendulum was characterized by the standard deviation of the time-series of the tilt angle θ, referred to as *SD*-θ. It can be determined not only by the intensity of motor noise (σ in the computational models), but also by the motor control strategy employed by the CNS, including the gains of the feedback controller and the parameter *a* that determines the switching boundary. Several types of intermittent control models exhibit larger fluctuations than the continuous feedback control model (Bottaro et al., 2008; Asai et al., 2009; Nomura et al., 2013), even with low noise.

### Stability analysis of the models and fitting experimental behaviors to the models

Each experimental time series of the tilt angle θ was fitted by three types of computational models (the continuous control model, the type-1 intermittent control model, and the type-2 intermittent control model) to determine which model, with an appropriate set of parameters, could best reproduce the experimental behavior. The goodness of the fit was quantified based on five indices that characterize the tilt angle fluctuation: the two non-Gaussianity indices $\lambda_{q\to 0}^{2}$ and $\lambda_{q=1}^{2}$, the ICM-median for the ICM distribution, the PSD-PF index for the PSD, and the SD-θ index for the tilt angle time-series. To this end, the dynamics of the models were simulated for a variety of parameter values for the set $\vec{P}\equiv \left(a,\;P_{\theta },D_{\theta },D_{x},\sigma ,type\right)$, where the examined parameter range was as follows: the value of *a* varies as $a=\sqrt{2g/\ell }\tan\varphi$ for varying φ ∈ [−π/2 + 0.0001, 9π/20 + 0.0001] with a step of π/20, *P*_θ_ ∈ [0, 15] with a step of 0.25, *D*_θ_ ∈ [0, 3] with a step of 0.15, *D*_*x*_ ∈ [0, 3] with a step of 0.15, σ ∈ [0.001, 0.031] with a step of 0.0025, and type ∈ [1, 2] representing the type of the intermittent control. The continuous control model with various *P*_θ_-*D*_θ_-*D*_*x*_ gains is included in the type-1 and type-2 intermittent control models as a special case with *a* = −∞.

The parameter-dependent dynamics of the models were examined to clarify how the five-dimensional index vector, denoted by $\vec{I}\left(\vec{P}\right)$, changed as a function of the parameter vector $\vec{P}$ for a simulated time-series of the model. It provides stability regions in the parameter space, as the five indices can be calculated only for models with parameter vectors $\vec{P}=\left(a,\;P_{\theta },D_{\theta },D_{x},\sigma ,type\right)$ that exhibit stable dynamics. Let ${\vec{I}}_{exp}=\;$($\lambda_{q\to 0}^{2},\;\lambda_{q=1}^{2},$ ICM-median, PSD-PF, *SD*-θ) be the five-dimensional index vector for a given experimental time-series. The parameter vector ${\vec{P}}_{best}$ that gives the best-fit-model in reproducing the experimental behavior for each subject was obtained as follows:

(20) $${\vec{P}}_{best}=\underset{\vec{P}}{\min}{\vec{I}}_{e}\;W\;{\vec{I}}_{e}^{\text{T}},$$

Where

$${\vec{I}}_{e}=\vec{I}\left(\vec{P}\right)-{\vec{I}}_{exp},$$

and

$$W=\text{Diag}(1/s_{1},\;1/s_{2},1/s_{3},1/s_{4},1/s_{5}),$$

with *s*_1_, *s*_2_, *s*_3_, *s*_4_, and *s*_5_ being the standard deviations of $\lambda_{q\to 0}^{2}$, $\lambda_{q=1}^{2}$, ICM-median, PSD-PF, and *SD*-θ, respectively, across all simulated time series for all examined parameter sets and initial conditions. That is, ${\vec{I}}_{e}W{\vec{I}}_{e}^{\text{T}}$ in Equation (20) represents the error function (or the fitness function) for measuring the distance between simulated and experimental data, i.e., $\vec{I}\left(\vec{P}\right)$ and ${\vec{I}}_{exp}$, based on the five parameters weighted by their standard deviations.

## Results

### Movement variability in the experimental task

Table 1 summarizes the largest (*d*_*max*_) and mean (*d*_*mean*_) values of the balancing-time duration for six subjects (Subjects 1 to 6) who qualified as skilled performers. The remaining six out of the 12 subjects did not satisfy the skilled-performer criterion within the limited period of motor learning, indicating the difficulty of the task. Based on the values for *d*_*max*_ and *d*_*mean*_, Subjects 1, 3, and 5 were considered as the most skilled performers. The durations *d*_*max*_ and *d*_*mean*_ were shortest for Subject 2, although Subject 2 qualified as a skilled performer.

**Table 1:** **Largest (*d*_*max*_) and mean (*d*_*mean*_) values of balancing-time duration for the skilled subjects (skilled performers; Subjects 1–6)**.

	**d_*max*_ (S)**	**d_*mean*_ (S)**	**# of trials**
Subject 1	558	530	2
Subject 2	297	263	3
Subject 3	600	453	2
Subject 4	369	293	3
Subject 5	500	408	2
Subject 6	464	283	3

*d_max_, the longest balancing-time duration for each skilled subject among all the trials. d_mean_, the mean value of the balancing-time duration over the skilled trials that were used for assembling the 10 60-s data segments. # of trials, the number of trials that was used for collecting the ten 60-s data segments. A longer balancing duration of the best and second- and third-best trials led to a smaller number of trials necessary for collecting the 10 data segments. See the definitions of d_max_ and d_mean_ in the main text*.

**Figure 3:**
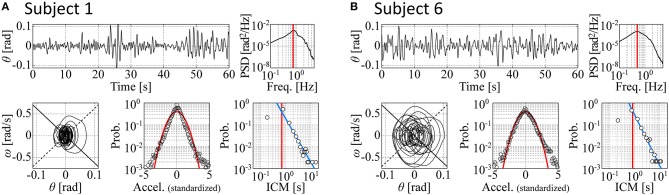
**Time-series data for two experimental subjects with the corresponding phase portraits, cart-acceleration distributions, PSD functions, and ICM distributions. (A,B)** are for Subjects 1 and 6, respectively. The data for each subject are a segmented piece (60 s). Top left, tilt angle; top right, PSD; bottom left, phase portrait with a piece of trajectory for a selected period of 20 s; bottom middle, cart-acceleration distribution; bottom right, ICM distribution. The red curves in the acceleration distribution represent the Gaussian. The vertical red lines in the ICM and PSD represent the values of two indices: the ICM-median and PSD-PF. See the text for details.

Figure 3 shows the time-series data for two subjects (Subjects 1 and 6) with the corresponding phase portraits, cart-acceleration distributions, PSD functions, and ICM distributions. The non-gray-shaded rows in Table 2 indicate the values of the five indices, i.e., ${\vec{I}}_{exp}=$ ($\lambda_{q\to 0}^{2},\;\lambda_{q=1}^{2},$ ICM-median, PSD-PF, *SD*-θ), for each of the six skilled subjects. Subject 1—one of the most skilled subjects as shown in Table 1—exhibited the second-largest non-Gaussianity index $\lambda_{q=1}^{2}$, which is consistent with the apparent fat tail in the acceleration distribution shown in Figure 3A. Regarding two other most skilled subjects (Subjects 3 and 5), the non-Gaussianity in terms of $\lambda_{q=1}^{2}$ was also large for Subject 3 but not as large for Subject 5. Nevertheless, the values of $\lambda_{q=1}^{2}$ deviated significantly from zero for all skilled subjects (see Figure 3B for Subject 6), meaning that movement variability for the skilled subjects in this task can be well characterized by the non-Gaussian acceleration distribution, as reported by previous studies (Cabrera and Milton, 2004a; Cluff and Balasubramaniam, 2009). The non-Gaussianity in terms of $\lambda_{q\to 0}^{2}$ was also large for all the skilled subjects, indicating that the acceleration distribution for each subject always exhibited leptokurtosis at the origin. Frequent appearances of the null acceleration imply that the subjects frequently did not control the motions of the cart.

**Table 2:**
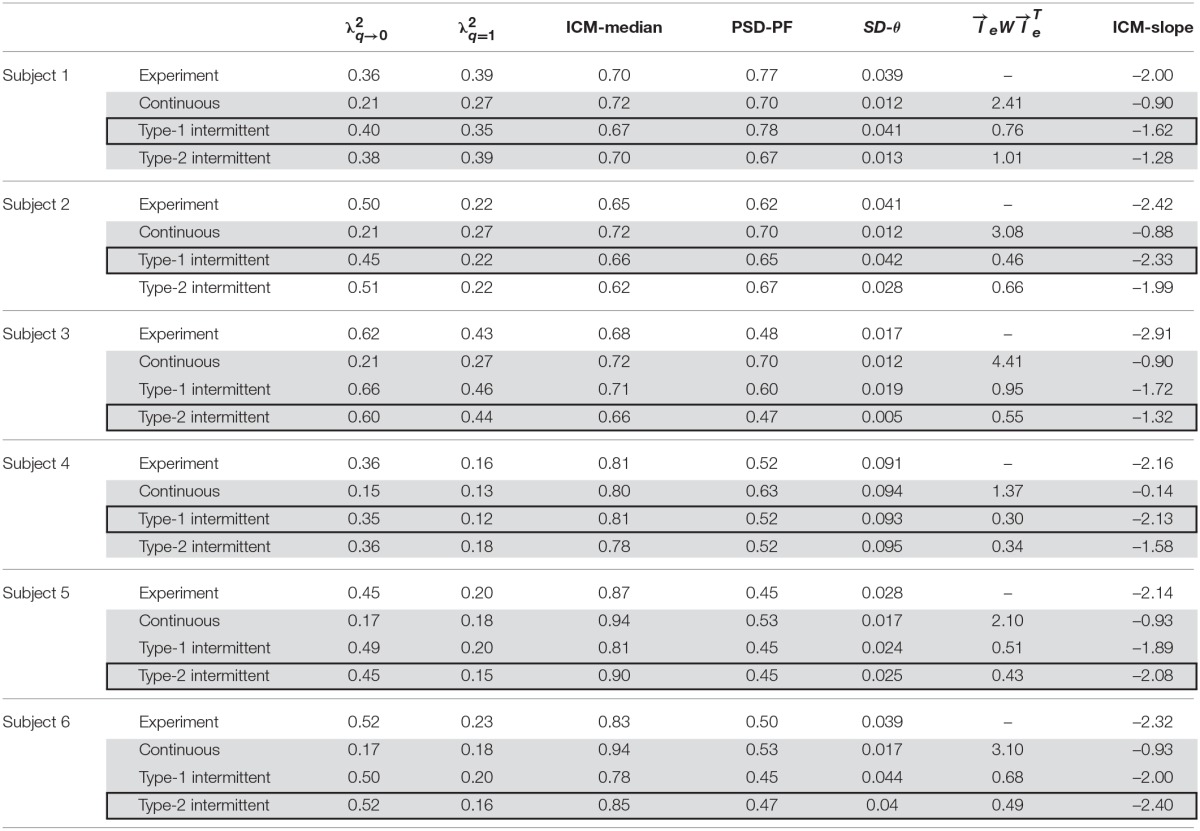
**Index values for characterizing movement variability**.

*For each subject, the values of five indices are summarized for the experimental data as well as for the simulated data with the best- fit-models of the continuous, type-1 intermittent, and type-2 intermittent control models. The best- fit-model among the three types of control models for each subject is highlighted by the thick-box. The second column from the right and the rightmost rows represent the values of the fitness (error) function defined in Equation (20) and the ICM-slope index values, respectively*.

The values of the two non-Gaussianity indices ($\lambda_{q=1}^{2}$ and $\lambda_{q\to 0}^{2}$) were similar for Subject 1 but not necessarily for the other subjects. This means that the acceleration distribution of Subject 1 was consistent with a multiplicative lognormal distribution (Equation 17). This was not necessarily true for the other subjects, although the acceleration distributions in all subjects deviated significantly from Gaussian.

The ICM-distributions for each subject exhibited power-law behaviors as reported by a previous study (Cabrera and Milton, 2002), with the scaling exponent values around 2.0 (the ICM-slope around –2.0 as shown in the right-most column of Table 2) that were not necessarily close to 3/2 as reported in Cabrera and Milton (2002). Indeed, the scaling exponents (and thus the ICM-slope values) were very sensitive to the choice of the threshold used to determine the onsets of the on-phases.

The trajectory in the θ-ω plane did not necessarily circulate simply around the origin, but they tended to be elongated horizontally, suggesting that the mechanisms associated with the hyperbolic trajectory within the off-regions and/or the alternating pseudo-equilibrium points are involved in the manual control of the subjects.

### Stability and movement variability in the computational models

**Figure 4:**
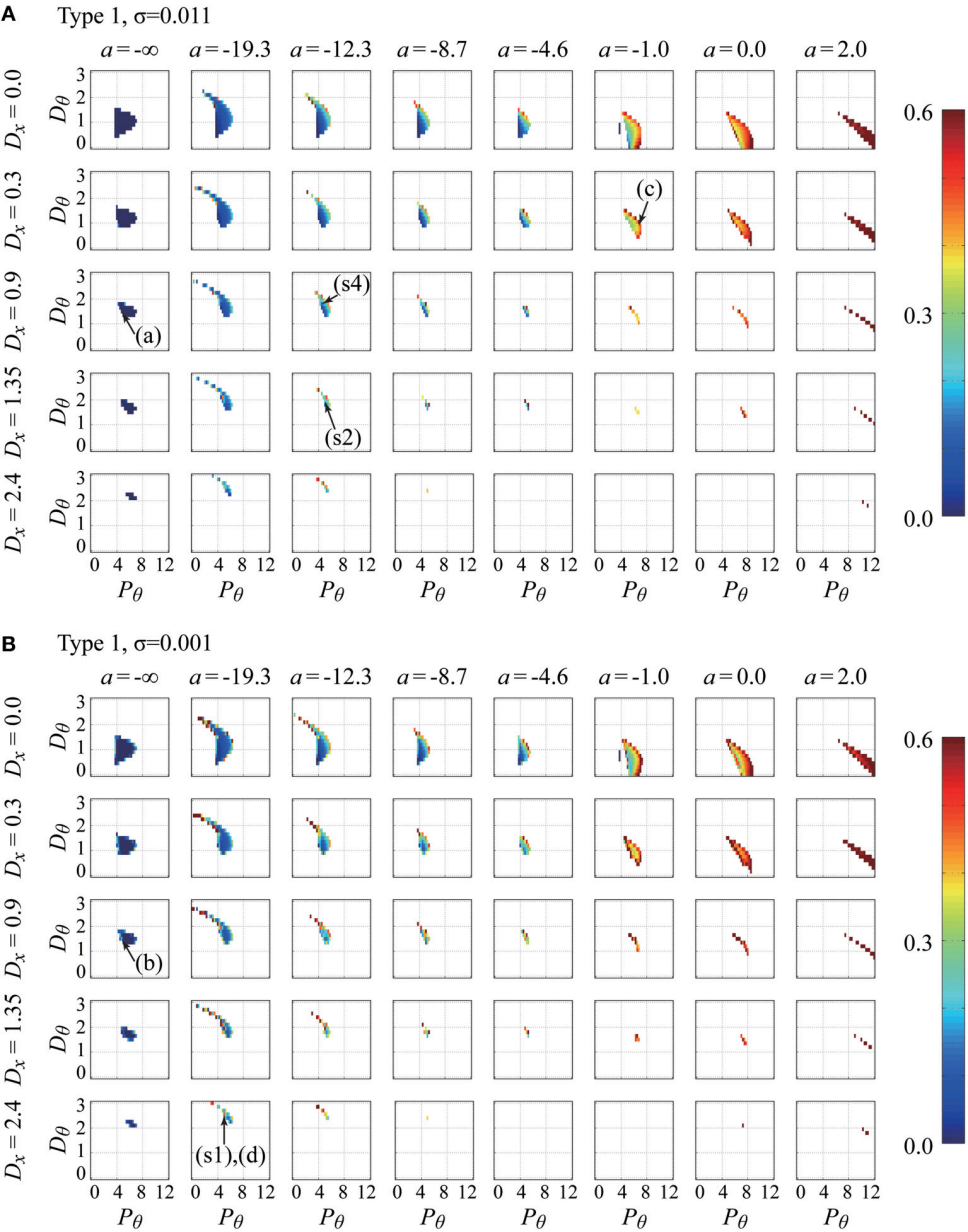
**Parameter dependency of stability and the non-Gaussianity index $\lambda_{q=1}^{2}$ for the movement variability of the pendulum in the type-1 intermittent feedback control models with two typical noise intensities (delay-time Δ = 0.1s)**. Values of the index $\lambda_{q=1}^{2}$ were calculated for the model with various parameter vectors $\vec{P}$ = (*a*, *P*_θ_, *D*_θ_, *D_x_*, σ, type = 1), which are plotted by color-code in the *P*_θ_-*D*_θ_ planes for a set of combinations of the values of *a* (columns) and *D*_*x*_ (rows). The noise intensity is σ = 0.011 for the upper sets **(A)** of the *P*_θ_-*D*_θ_ planes and σ = 0.001 for the lower sets **(B)** of the *P*_θ_-*D*_θ_ planes. Because the index values can be calculated only when the models exhibit stable dynamics, the colored regions also represent the stability regions of the model. The parameter points indicated by the arrows with **(A–D)** are used for generating the time-series shown in Figures 5A–D, respectively. Those with (s1), (s2), and (s4) are the parameter points that best fit the experimental behaviors for Subjects 1, 2, and 4, respectively. Note that the best-fit noise intensities for (s1), (s2), and (s4) are not the values used for these panels (see Table 3 for the exact values). See the text for details.

**Figure 5:**
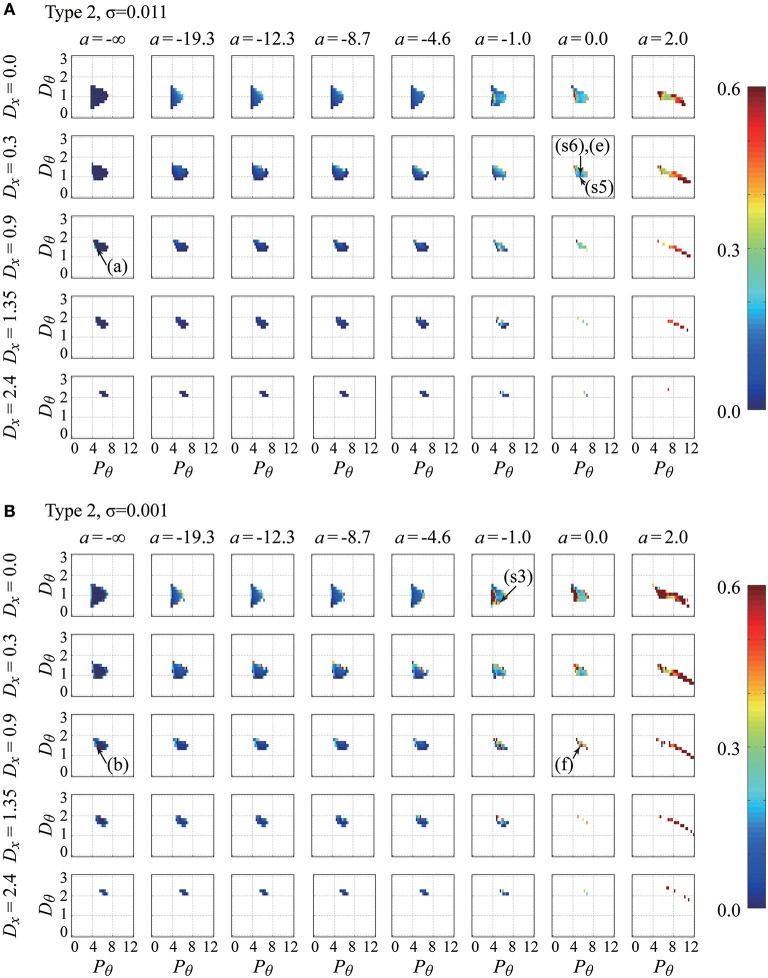
**Parameter-dependency of stability and the non-Gaussianity index $\lambda_{q=1}^{2}$ for the movement variability of the pendulum in the type-2 intermittent feedback control models with two typical noise intensities (delay-time Δ = 0.1 s)**. The figure is arranged in the same way as Figure 4. The noise intensity is σ = 0.011 for the upper sets **(A)** of the *P*_θ_-*D*_θ_ planes and σ = 0.001 for the lower sets **(B)** of the *P*_θ_-*D*_θ_ planes. The parameter points indicated by arrows with **(A**,**B,E,F)** are used for generating the time-series shown in Figures 6A,B,E,F, respectively. Those with (s3), (s5), and (s6) are the parameter points that best fit the experimental behaviors for Subjects 3, 5, and 6, respectively. Note that the best-fit noise intensities for (s3), (s5), and (s6) are not the values used for these panels (see Table 3 for the exact values). See the legend of Figure 4 and the main text for details.

Figures 4, 5 show the parameter dependency of the non-Gaussianity index $\lambda_{q=1}^{2}$ for the movement variability of the pendulum in the type-1 intermittent and type-2 intermittent feedback-control models, respectively, with two typical noise intensities for each model. Because the index $\lambda_{q=1}^{2}$ can be calculated only for the models with parameter vectors $\vec{P}=\left(a,\;P_{\theta },D_{\theta },D_{x},\sigma ,type\right)$ that exhibit stable dynamics, Figures 4, 5 also represent the stability regions of the models. That is, the regions indicated by colors denote stability irrespective of the color. Figure 4 shows how the non-Gaussianity index $\lambda_{q=1}^{2}$ changes as values of *a*, *P*_θ_, *D*_θ_, and *D*_*x*_ change for the type-1 intermittent control model with parameter vectors $\vec{P}=\left(a,\;P_{\theta },D_{\theta },D_{x},0.001,1\right)$ with a small noise intensity (upper panels), and with $\vec{P}=\left(a,\;P_{\theta },D_{\theta },D_{x},0.011,1\right)$ with a medium noise intensity (lower panels). Similarly, Figure 5 provides corresponding information for the type-2 intermittent control model with parameter vectors $\vec{P}=\left(a,\;P_{\theta },D_{\theta },D_{x},0.001,2\right)$ and $\vec{P}=\left(a,\;P_{\theta },D_{\theta },D_{x},0.011,2\right).$ Each figure consists of a number of panels corresponding to the *P*_θ_−*D*_θ_ parameter planes spanned by the proportional and derivative gain parameters in the range of *P*_θ_ ∈ [0, 12.0] and *D*_θ_ ∈ [0, 3.0]. The model for a given set of *P*_θ_-*D*_θ_ parameter values is represented by the corresponding grid in the *P*_θ_-*D*_θ_ plane, and the colored grid indicates that the model with that parameter values exhibits stable dynamics. For each parameter set for stable dynamics, a set of values for the index vector $\vec{I}$ (*P*_θ_, *D*_θ_) was calculated, and particularly the value of the non-Gaussianity index $\lambda_{q=1}^{2}$ is depicted using the color-code as a function of *P*_θ_ and *D*_θ_ in Figures 4, 5. In Figures 4, 5, for the small and medium noise intensities, the *P*_θ_ − *D*_θ_ planes in the first column (leftmost column) are for *a* = −∞, meaning that those *P*_θ_-*D*_θ_ planes represent the stability regions of the continuous control model with different values of the gain parameter *D*_*x*_.

In Figures 4, 5, one can observe that the stability region is roughly located at the middle of each *P*_θ_ − *D*_θ_ plane. The stability region in the *P*_θ_-*D*_θ_ plane changes based on the other parameters. For a given value of a (for a given column), the stability region diminishes as the gain parameter *D*_*x*_ increases, indicating that the feedback controller for the cart velocity decreases stability. For *a* = −∞ with *D*_*x*_ = 0 (top left corner of Figures 4, 5), the lower bound of *P*_θ_ (the critical proportional gain *P*_θ*cr*_) is 3.75 N/rad, which is consistent with the theoretical value (*P*_θ*cr*_ = 3*mg* = 3.679 N/rad), and it shifts to the right as *D*_*x*_ increases. For small fixed values of *D*_*x*_, the stability region changes substantially as *a* increases. In particular, for the set of the *P*_θ_-*D*_θ_ planes arranged in the first and second rows of Figure 4 for the type-1 intermittent control model, as a increases from −∞ toward zero and then to positive values, the stability region is stretched in the direction of small *P*_θ_ (and large *D*_θ_) values below the critical proportional gain *P*_θ*cr*_ and then stretched in the direction of large *P*_θ_ (and small *D*_θ_ close to zero). For large positive a values, the stability region becomes small and eventually diminished as *a* increases. As shown in Figure 5, for the type-2 intermittent control model, the stability region does not change significantly until a becomes close to zero, and then it is elongated horizontally in the direction of large *P*_θ_ values for positive a values as *a* increases. A comparison between the upper (medium noise) and lower (small noise) sets of panels indicates that the intensity of additive noise does not change the stability region but does change the color of the stability regions (the non-Gaussianity).

The color spectrum from dark blue to dark red is used for representing the non-Gaussianity index $\lambda_{q=1}^{2}$ shown in Figures 4, 5, where the most dark blue and red represent $\lambda_{q=1}^{2}=0.0$ and $\lambda_{q=1}^{2}=0.6$ or larger than 0.6 (the largest value exhibited by the simulated time-series data for the examined parameter range was 2.27), respectively. In Figures 4, 5, for a given value of *D*_*x*_, the color of the grid (color of the stability region) changes from blue to light blue and then from yellow to red as a increases from *a* = −∞ to zero and then to positive values, e.g., *a* = 2.0 (in particular for the set of the *P*_θ_-*D*_θ_ planes arranged in the first to the third rows), indicating that the non-Gaussianity increases as the off-regions become wide. That is, large off-regions are responsible for the increasing non-Gaussianity in the movement variability. The stability regions in the *P*_θ_-*D*_θ_ planes arranged in the first column of Figures 4, 5, whose colors represent the non-Gaussianity of the continuous control models, are mostly colored blue, meaning that movement variability of the continuous control models can rarely exhibit non-Gaussianity, and the corresponding acceleration distribution is typically Gaussian. Moreover, for each colored stability region in a given *P*_θ_-*D*_θ_ plane, there is a tendency that the color is more red shifted at the stability boundary, i.e., at the edge of each stability region, than at the center of stability region, indicating that the criticality at the stability boundary may be associated with the degree of the non-Gaussianity. Interestingly, this is also the case even for the continuous control model but only with *D*_*x*_ > 0 involving the pseudo-equilibrium point and with small noise intensity (see carefully the first column of Figures 4B, 5B with *D*_*x*_ > 0).

**Figure 6:**
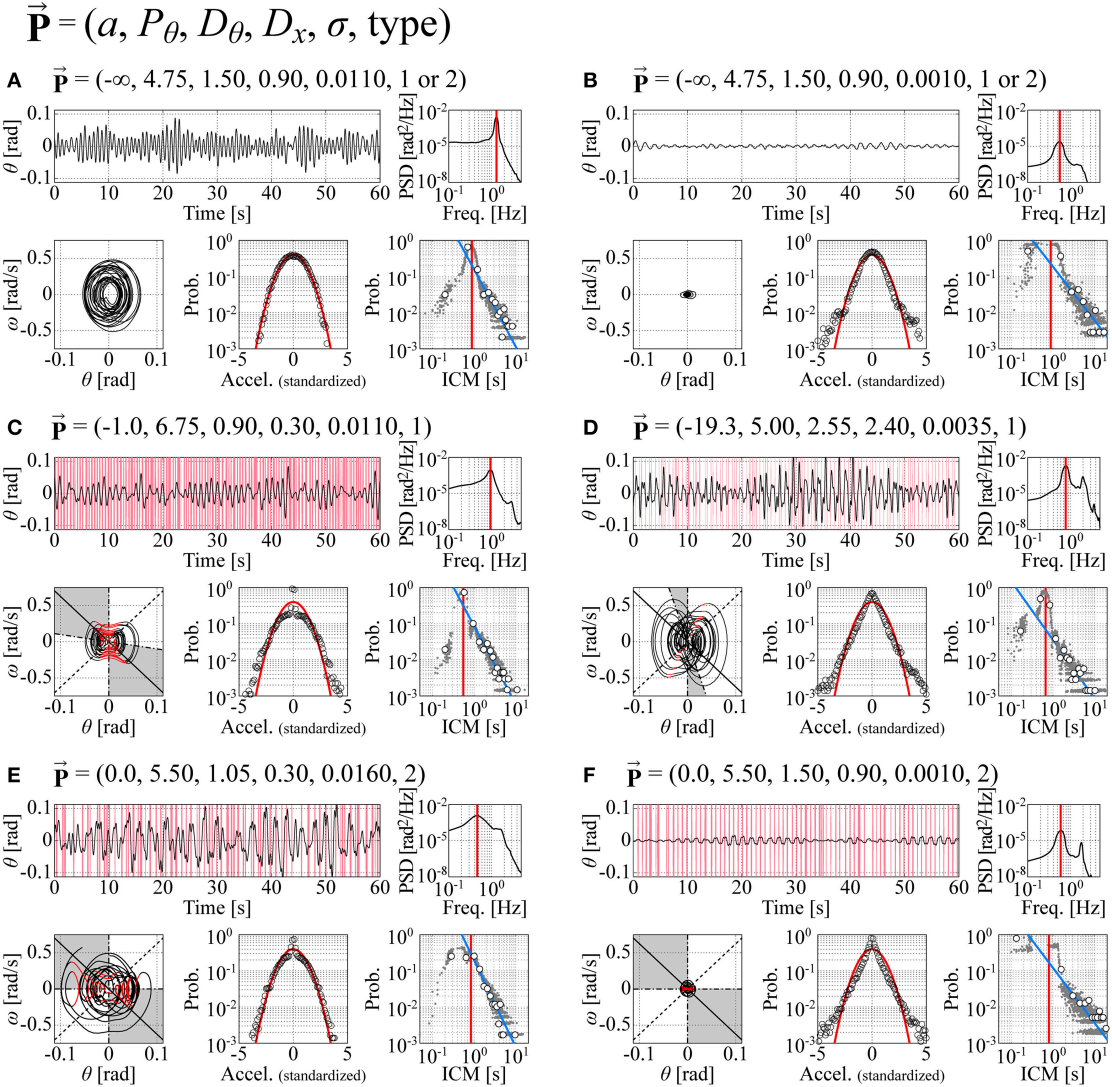
**Typical time-series data of the models with their corresponding phase portraits, cart-acceleration distributions, PSD functions, and ICM distributions**. Panels are prepared for six sets of the parameter vectors $\vec{P}$ indicated at the top of each trace. The configuration in each of the **(A**–**F)** is exactly the same as in Figure 3. Each ICM distribution includes a number of small dots that were obtained from 100 simulated sample paths with a duration of 60 s. **(A,B)**: Continuous feedback control models. **(C,D)**: Type-1 intermittent control models. **(E,F)**: Type-2 intermittent control models. See the legend of Figure 3 and the text for details. Dynamics in the **(D,E)** best fit the experimental behaviors of Subject 1 (Figure 3A) and Subject 6 (Figure 3B), respectively. For the intermittent control models in the **(C**–**F)**, red vertical lines in the θ-waveforms represent the off-phases during which the active feedback control is switched off, and the black and red curves in the phase portraits represent the trajectories during the on-phases and off-phases, respectively.

Figure 6 exemplifies typical time-series data of the models with their corresponding phase portraits, cart-acceleration distributions, PSD functions, and ICM distributions, in which panels are prepared for the following six sets of parameter vectors $\vec{P}$: ${\vec{P}}_{cont}^{m}$ = (−∞, 4.75, 1.5, 0.9, 0.011, 1 or 2) with a medium noise intensity (Figure 6A), ${\vec{P}}_{cont}^{s}$ = (−∞, 4.75, 1.5, 0.9, 0.001, 1 or 2) with a small noise intensity (Figure 6B), ${\vec{P}}_{type1}^{m}=\left(-1.0,6.75,0.9,0.3,0.011,1\right)$ with a medium noise intensity (Figure 6C), ${\vec{P}}_{type1}^{s}=\left(-19.3,5.0,2.55,2.4,0.0035,1\right)$ with a small noise intensity (Figure 6D), ${\vec{P}}_{type2}^{m}=\left(0.0,5.50,1.05,0.3,0.016,2\right)$ with a medium noise intensity (Figure 6E), and ${\vec{P}}_{type2}^{s}=\left(0.0,5.5,1.5,0.9,0.001,2\right)$ with a small noise intensity (Figure 6F). The parameter vectors $\vec{P}$ for the panels (**A–F**) in Figure 6 are located in the parameter space in Figures 4, 5, as indicated by the corresponding symbols. The movement variability in the type-1 and type-2 intermittent control models exhibits non-Gaussian acceleration distribution and a power-law-like ICM distribution in a range of ICM intervals longer than ~0.8 s.

The trajectory in the θ-ω plane for the continuous model with a medium noise intensity is not affected by the pseudo-equilibrium point, and thus it circulates almost around the origin (Figure 6A). However, when the *P*_θ_-*D*_θ_ parameter values of the model are set very close to the stability boundary and the noise intensity is small, the right-left alternating pseudo-equilibrium point causes a trajectory of the butterfly-wings-like shape (confirmed by magnifying Figure 6B), leading to the non-Gaussian acceleration distribution (Figure 6B). See the light-blue-colored small grid indicated by “**(B)**” in the first column of Figures 4, 5 (although the color is not visible due to the small grid size). The trajectory for the type-1 intermittent control model exhibits a peanut-like shape even with a small *D*_*x*_ value and a medium noise intensity, for which the hyperbolic curve segments during the off-phase (red segments of the trajectory in Figures 6C) are responsible, leading to the non-Gaussian acceleration distribution particularly at the origin with the peaky-shape (the large kurtosis). The type-1 intermittent control model with a large *D*_*x*_ value exhibits an apparently butterfly-wings-shaped trajectory (Figure 6D), for which the combination of the hyperbolicity in the off-phases and the right-left alternating pseudo-equilibrium point responsible, leading to the perfectly lognormal-shaped acceleration distribution with the peaky-shape at the origin and the fat tail. The type-2 intermittent control model can be characterized similarly. Notably, the type-2 intermittent control model can exhibit a butterfly-wings-shaped trajectory and a non-Gaussian acceleration distribution even with a relatively small *D*_*x*_ value (Figure 6F), for which the _θ_Δ_*v*Δ_-dependent onset of the off-phase and the _θ_Δ_ ω Δ_-dependent onset of the on-phase, rather than the pseudo-equilibrium point are responsible.

### Fitting the experimental movement variability to the models

Table 2, with gray-shaded rows, presents the values of the five indices, i.e., $\vec{I}=$ ($\lambda_{q\to 0}^{2},\;\lambda_{q=1}^{2},$ ICM-median, PSD-PF, *SD*-θ) and the minimized fitness function (${\vec{I}}_{e}W{\vec{I}}_{e}^{\text{T}}$) for each of the three types of control models that exhibits an index vector closest to the experimental index vector ${\vec{I}}_{exp}$ for each of the six skilled subjects. The movement variabilities of the pendulum in all subjects were best fitted by the intermittent control models, as indicated by the rows with thick boxes in Table 2. Three out of the six skilled subjects were best fitted by the type-1 intermittent control models, and the remaining three were best fitted by the type-2 intermittent control models. The values of the fitness function for the type 1 and 2 intermittent control models are small and relatively close to each other. However, the values of the fitness function for the continuous control model are always remarkably larger than those for the intermittent control model, indicating that the continuous control model could hardly exhibit behaviors similar to the experimental data.

Table 3 summarizes, for each of the three types of control models, the values of the parameter vector ${\vec{P}}_{best}$ that gives an index vector $\vec{I}$ closest to the experimental index vector ${\vec{I}}_{exp}$ for each of the six skilled subjects. The locations of the six ${\vec{P}}_{best}$ vectors for subjects 1, 2, …, and 6 are roughly indicated by the symbols (s1), (s2), …, and (s6) in Figures 4, 5. The typical behaviors of the best- fit-models are exemplified in Figure 6. The movement variability for Subject 1 (Figure 3A) is best fitted by the type 1 intermittent control model, as depicted in Figure 6D. The movement variability for Subject 2 (Figure 3B) is best fitted by the type 2 intermittent control model, as depicted in Figure 6E. Interestingly, all of the six ${\vec{P}}_{best}$ vectors are located near the stability boundary, indicating that the movement variability of those best-fit-models are influenced by the criticality at the edge of stability.

**Table 3:**
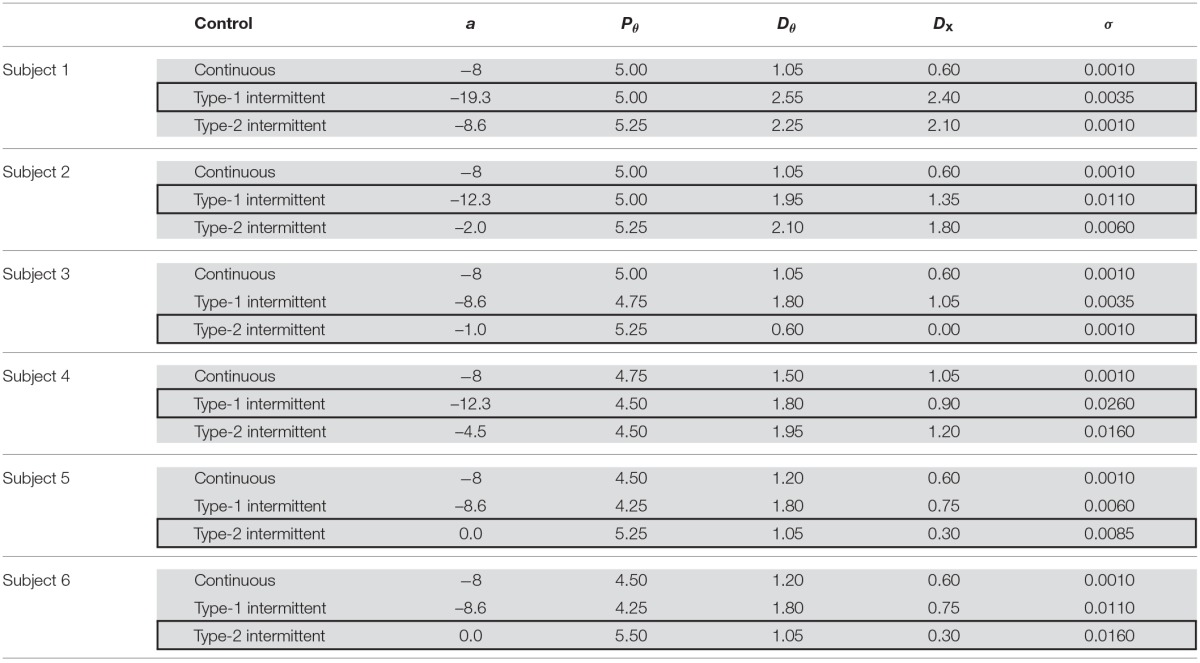
**Parameter values of the best-fit-model for each of the three types of control models**.

*For each subject, the values of five parameters for the best-fit-models of the continuous, type-1 intermittent, and type-2 intermittent control models are summarized. The best-fit-model among three types of control models for each subject is highlighted by the thick box*.

## Discussion

We attempted to demonstrate that a motor control strategy for stabilizing an inverted pendulum on a manually controlled cart (i.e., CIP), analogous to stick balancing on the fingertip, can be characterized by intermittent time-delay feedback control (Bottaro et al., 2008; Asai et al., 2009, 2013). We measured the experimental behaviors concerning CIP dynamics for experimental subjects while they performed a balancing task. In particular, the movement variability during a CIP task for the skilled subjects was quantified using several summary measures, including (1) the non-Gaussianity of distributions of the cart-acceleration, (2) the distribution of the time intervals of two successive corrective movements, (3) the characteristic peak frequency in the power spectra for the motion of the pendulum, and (4) the standard deviation for the motion of the pendulum. We showed that in all the subjects who could acquire skillful motor performance for the task, (1) the non-Gaussianity in the cart-acceleration distributions increased, (2) the distribution of the inter-corrective movement intervals tended to exhibit power-law-like behaviors with a larger probability for long inter-corrective movements, and (3) the characteristic peak in the power spectra of the motion of the pendulum was located in the low-frequency band. These results are consistent with previous reports (Cabrera and Milton, 2002, 2004a).

Computational models that can reproduce the experimental CIP behaviors provide mechanistic interpretations of how the experimental subjects stabilized the inherent unstable dynamics of the CIP. We showed that the CIP dynamics for all skilled subjects could be better fitted by the models with the intermittent time-delayed feedback controller than by those with the continuous time-delayed feedback controller, suggesting that the human CNS stabilizes the upright posture of the pendulum by utilizing the intermittent control strategy. The experimental behaviors for half of the skilled subjects were best fitted by the intermittent feedback control model (Type-1), and those for the remaining half of the subjects were best fitted by the intermittent feedback control model (Type-2).

### Stabilization strategy to overcome the risk of falls

The CIP dynamics and the corresponding corrective movements for the skilled subjects can be summarized as follows: skilled subjects correct the cart movement less frequently; i.e., they do not interfere with the motion of the pendulum when “the risk of falls” is small, which generates long inter-corrective movements and a peaky shape in the acceleration distribution with a large kurtosis around the origin (null-accelerations). On the other hand, they intervene in the CIP dynamics more frequently when “the risk of falls” becomes larger than expected by Gaussian-type statistics, which generates a fatter tail in the acceleration distribution than that in the Gaussian distribution in the large-acceleration regime. These experimental behaviors can be interpreted by the intermittent control models. In both types of the intermittent control models, the feedback controller is turned off when the state of the pendulum is close to the stable manifold in the θ-ω plane. Typically, the corresponding off-phases take place when the cart and the pendulum go by each other with the opposite directions (i.e., the signs of the cart velocity and the pendulum velocity are opposite “physically”), as illustrated in the θ-*v* plane of Figures 2C–F where the red curve segments tend to appear in the first and third quadrants of the θ-*v* plane with θ approaching the origin.

In the type-1 intermittent control model, the slow dynamics of the pendulum moving from the left (right) side of the θ-ω plane along the stable manifold to the right (left) side of the θ-ω plane along the unstable manifold during off-phases as shown by the red curve segments in Figures 2C,D, **6C**, which are responsible for generating the long inter-corrective-movements and also for the non-Gaussianity of the acceleration distribution around the origin. The slow dynamics of the pendulum (as illustrated by the red curve segments in Figure 2D can appear in a different way in association with the pseudo-equilibrium point that appears on the θ-axis in the θ-ω plane alternately either the right- or the left-hand-side of the origin when the delayed feedback controller with respect to the cart velocity (Equation 5) is turned on during on-phases. Although the state of the pendulum circulates once or several times around the pseudo-equilibrium point even in the continuous control model, a part of the circulating trajectory becomes control-free dynamics because the circulating trajectory often overlaps substantially with the off-regions in the type-1 intermittent control model (see the red curve segments in Figures 2D, 6D. The intermittent appearances of the slow dynamics with these two processes generate a peanut-shaped and/or a butterfly-wings-shaped trajectory in the θ-ω plane, characterizing the skilled performance. This implies that the risk of falls is small when the system exhibits the slow dynamics along the red curve segments, and a big risk of falls does not necessarily imply a large tilt-angle of the pendulum. Interestingly, the peanut and/or the butterfly-wings-shaped trajectory is invariant over the oscillation amplitude around the origin. This invariance implies that the feedback controller and thus also the skilled subjects, probably, manipulate the cart in the same way regardless of the size of the risk and the size of the interventions (accelerations), leading to the power-law distribution in the inter-corrective movement intervals.

The type-2 intermittent control model with the cart-velocity-dependent off-on switching conditions also exhibits the butterfly-wings-shaped trajectory (v 2(e)-(f) and Figures 6E,F) by using the pseudo-equilibrium point during the on-phase. However, it is less apparent than the type-1 model, because the pendulum in the type-2 model passes though the upright position during on-phases in contrast to the type-1 model, which makes the trajectories moving from one side of the θ-ω plane to the other side of the θ-ω plane round (the black arc segments lying between the right and the left half planes) as distinct from the hyperbolic curves (the red curve segments lying between the right and the left half planes) in the type-1 model. In other words, the tilt angle of the pendulum in the type-2 model is altered from side to side actively with the feedback force, whereby the pendulum tends to gain a large falling velocity after it passes through the upright position. The resultant fast dynamics, in contrast to the slow dynamics along the stable and unstable manifolds for the type-1 model, should be halted quickly by reversing the cart motion (i.e., by generating a large control force with a large cart acceleration), after which the large control force should be terminated immediately for triggering an off-phase with slow dynamics, if not the pendulum falls. The skillfulness [modeled by the switching conditions in Equation (16)] enables the subjects (the feedback controller model) to achieve this quick transition between the on-phase and the off-phase.

### Similarity between the pseudo-equilibrium point and ballistic-bias control model

The delayed intermittent feedback-controller examined in this study stabilizes the pendulum in a similar way as the one proposed for the human postural control during quiet standing (Bottaro et al., 2008; Asai et al., 2009, 2013) in the sense that both controllers exploit the slow dynamics along the stable manifold of the upright saddle point during the off-phases. Meanwhile, the appearance of the pseudo-equilibrium point during the CIP stabilization, which moves alternately from side to side on the θ-ω plane for the CIP controller involving the derivative feedback control with respect to the cart velocity, characterizes the CIP system differently from the human postural control in which the ankle joints (corresponding to the pivot of the stick) are fixed in the space.

Interestingly, however, dynamics of the state point that chases the pseudo-equilibrium point in an anti-phase manner in the intermittent control model for the CIP seems similar conceptually to the ballistic bias control model proposed for the human postural control (Loram and Lakie, 2002; Lakie et al., 2003; Loram et al., 2005). In the bias control model, the CNS controls “the bias” that plays a role of the virtual equilibrium-point trajectory that has been considered during human arm reaching movement (Gomi and Kawato, 1996), where the mechanical system (e.g., the arm or the standing body) chases the virtual equilibrium-point trajectory as the desired trajectory. A large bias, i.e., a large difference between the current state and the desired state, is generated actively by the CNS in a feedforward and/or anticipatory manner in order to generate an appropriate control force even with a small stiffness of the joint actuator (a set of the antagonist muscles actuating the joint). In the intermittent control model for the CIP, re-positioning of the cart, particularly reverse in the direction of the cart movement, is performed based on the delayed information, in which the pendulum and the cart moves in an anti-phase manner. That is, the cart always chases the pendulum and catches it up, and then chases it again repeatedly. The pseudo-equilibrium point alternating from side to side represents such chase-and-escape dynamics, and the cart velocity that might reflect a distance between the chaser and the escaper, i.e., the bias, creates the pseudo-equilibrium point. Although the model examined in this study considered only the feedback controller, well-skilled subjects might control the cart position (also velocity and acceleration) in an anticipatory manner, leading to a more skilled performance of the task.

### Criticality at the edge of stability

Interestingly, the set of parameters of the models for the best-fit intermittent control models were located relatively close to the stability boundary of the models. The results imply that the mechanisms for non-Gaussian and power-law behaviors of the CIP dynamics are not necessarily limited to the intermittent feedback control and could be related to criticality of the system near the border of instability. This observation may be associated with the mechanism of “noise-induced stabilization” discussed by previous studies (e.g., Cabrera and Milton, 2002), in which a state-dependent multiplicative motor noise arises when the delay-affected system is tuned at, or near, the edge of stability and a critical control parameter is stochastically forced back and forth across the boundary.

Moreover, this observation might also be associated with a problem in the experimental estimate of the critical length where the stick always falls: sometimes it falls quickly other times it takes a longer time before it falls. This fact is consistent with a feedback controller whose parameters are tuned toward the edge of stability (Cabrera and Milton, 2004b). It might be useful to mention that, in delay equations models with state-dependent intermittent feedback such as considered in this study, there is a possibility that dynamics of the models include transient micro-chaos (Insperger et al., 2015). Although the current study deals with a simple scalar model of balancing task, it is likely that similar phenomena occur for more realistic models.

### Comparison of stability robustness among the models

The stability regions for the type-1 and type-2 intermittent control models were not necessarily significantly wider than those for the continuous control model, as shown in Figures 4, 5. This is not consistent with the cases for the models of posture control during quiet standing with a single or double inverted pendulum (Asai et al., 2009; Suzuki et al., 2012), where the stability regions of the intermittent control model are far wider than those of the continuous control model. However, the stability region for the type-2 intermittent control model with positive values of α is widely extended for large values of the proportional gain *P*_θ_ (figures not shown). This means that stability of the CIP dynamics is less sensitive to the gain *P*_θ_, but the timings for triggering the off-phases and/or the on-phases are critical. Moreover, if we speculate a situation where the CNS can change the on-off switching boundary (i.e., the parameter *a* in the intermittent control models) adaptively depending on the risk of falls, the sum of all the stability regions across a variety of values of *a* can be considered as the stability region of the CIP system with the intermittent controller, which makes the overall stability region quite large, contributing to increasing the robustness of stability of the intermittent control model.

In addition to the computational study reported in this paper, we numerically explored the stability of the CIP dynamics in the continuous and the intermittent control models when the length of the pendulum is shorter than the critical length (ℓ_*cr*_ = 0.392 m), assuming Δ = 0.2 s for the same parameter range as examined in this study (results and figures not shown here). As theoretically predicted, we confirmed that there is no stability region in the continuous control model. However, remarkably, there are non-negligible stability regions in the type-1 intermittent control model, suggesting the robustness of the intermittent control model for CIP tasks with higher difficulty. Interestingly, one of our experimental subjects (Subject 3, one of the most skilled subjects) successfully balanced the CIP with a length of ℓ = 0.25 m for more than 70 s several times, which can be considered as an extremely skillful performance. Because this length is far shorter than the critical length, there is no possibility that the subject used the continuous feedback control with tuned gain parameters, which may support the fact that the CNS utilizes more sophisticated control mechanisms than the “tuned continuous time-delayed feedback control.”

It is interesting to point out that stick balancing on the fingertip or cart is a voluntary motor skill, and hence practice is required to both attain and maintain the skill. The phrase “road to automatic” has been used to emphasize that novices likely use state-dependent feedback control, and as skill is acquired, it moves onto progressively more efficient control strategies that might involve various degrees of anticipation, etc (for a review see, for example, (Milton et al., 2004)). Thus, it is not hard to imagine that there might be an evolution of the control strategies through state dependent feedback involving first position, then position/velocity then position/velocity/acceleration (Insperger et al., 2012) then the intermittent type of control described in this paper and by Cabrera and Milton (2002), and then finally to something even more sophisticated as the nervous systems learns more and more about the task. In other words intermittent control might just be a step on the road to automatic and the observation of our balancer who could balance a shorter stick might just be a lighthouse.

### Limitations of the current study

We considered only two types of computational models for fitting the experimental data. As mentioned in Introduction, several types of neural control mechanisms, including time-delayed feedback with multiplicative noise (Cabrera and Milton, 2002), model predictive controllers with a sensory uncertainty (Mehta and Schaal, 2002; Gawthrop et al., 2011; Loram et al., 2011; Insperger and Milton, 2014), act-and-wait control (Insperger and Stepan, 2010), and time-delayed feedback with proportional-derivative-acceleration (Insperger and Milton, 2014), can reproduce at least some aspects of the CIP dynamics. The current study alone cannot exclude the plausibility of those other mechanisms. Nevertheless, intermittent time-delay feedback control that exploits stable manifold and hyperbolic dynamics around the saddle point is a promising alternative strategy for how the CNS stabilizes unstable dynamics.

The models considered in this study assume enough rail length for the translational motion of the cart. However, in reality, the length of the rail is limited. Thus, the cart moves only within a given limited range and collides with either end of the linear track (which is also the case in the fingertip stick balancing, where the hand exceeds the range that the subjects can reach). Therefore, the CIP task requires the control of not only the rotational motion of the inverted pendulum but also the translational motion (position) of the cart. The inclusion of additional feedback controllers such as a proportional control for restoring the cart position back to the origin is beneficial for modeling this situation. However, in this study, the use of proportional feedback control for the cart position in the models examined substantially narrowed the stability region. This suggests that other control strategies than the intermittent proportional and derivative control, such as feedback controllers that utilize acceleration and/or higher derivative information (Insperger et al., 2012; Insperger and Milton, 2014) and predictive feedforward controllers (Mehta and Schaal, 2002; Gawthrop et al., 2011; Loram et al., 2011) may be exploited by the CNS.

## Author contributions

Designed research: NY, TN. Experimental data acquisition: NY. Mathematical model formulation, analysis and simulation: NY, YS. Time-series data analysis: NY, KK. Wrote paper: NY, YS, KK, and TN.

## Funding

This work was supported in part by JSPS grants-in-aid 26242041 and 26750147, MEXT HPCI project (Theme 3).

### Conflict of interest statement

The authors declare that the research was conducted in the absence of any commercial or financial relationships that could be construed as a potential conflict of interest.

## Footnotes

^1^Equation (17) represents the probability density function (PDF) of a random variable *X* = *W*exp*Y*, where *W* and *Y* are two independent Gaussian random variables with (mean, variance) of (0,1) and (0, λ^2^), respectively. For a stochastic process of *X* parameterized by time, the amplitude of the process determined by *X* is modulated according to exp *Y*. The PDF of *X* can be written as,

$$P\left(x\right)=\int_{0}^{\infty }\frac{1}{\sigma }P_{W}\left(\frac{x}{\sigma }\right)P_{Y}\left(\ln\sigma \;\right)d\left(\ln\sigma \;\right),$$

where *P*_*W*_ and *P*_*Y*_ are the PDFs of *W* and *Y*, respectively. If we take Gaussian for *P*_*W*_ and *P*_*Y*_, and express λ -dependeny of *Y* explicitly, we have Equation (17). Note that, as λ^2^ → 0, *W exp Y* → *W* and *X* becomes the standard Gaussian distribution.

## References

[B1] Asai Y. Tasaka Y. Nomura K. Nomura T. Casadio M. Morasso P. (2009). A model of postural control in quiet standing: robust compensation of delay-induced instability using intermittent activation of feedback control. PLoS ONE 4:e6169. 10.1371/journal.pone.0006169 19584944 PMC2704954

[B2] Asai Y. Tateyama S. Nomura T. (2013). Learning an intermittent control strategy for postural balancing using an EMG-based human-computer interface. PLoS ONE 8:e62956. 10.1371/journal.pone.0062956 23717398 PMC3661733

[B3] Bottaro A. Yasutake Y. Nomura T. Casadio M. Morasso P. (2008). Bounded stability of the quiet standing posture: an intermittent control model. Hum. Mov. Sci. 27, 473–495. 10.1016/j.humov.2007.11.005 18342382

[B4] Cabrera J. L. Milton J. G. (2002). On-off intermittency in a human balancing task. Phys. Rev. Lett. 89:158702. 10.1103/physrevlett.89.158702 12366030

[B5] Cabrera J. L. Milton J. G. (2004a). Human stick balancing: tuning Lévy flights to improve balance control. Chaos 14, 691–698. 10.1063/1.1785453 15446980

[B6] Cabrera J. L. Milton J. G. (2004b). Stick balancing: on-off intermittency and survival times. Nonlinear Stud. 11, 305–317.

[B7] Castaing B. Gagne Y. Hopfinger E. J. (1990). Velocity probability density functions of high Reynolds number turbulence. Physica D 46, 177–200. 10.1016/0167-2789(90)90035-N

[B8] Cluff T. Balasubramaniam R. (2009). Motor learning characterized by changing Lévy distributions. PLoS ONE 4:e5998. 10.1371/journal.pone.0005998 19543399 PMC2695787

[B9] Gawthrop P. Loram I. Lakie M. Gollee H. (2011). Intermittent control: a computational theory of human control. Biol. Cybern. 104, 31–51. 10.1007/s00422-010-0416-4 21327829

[B10] Gomi H. Kawato M. (1996). Equilibrium-point control hypothesis examined by measured arm stiffness during multijoint movement. Science 272, 117–120. 10.1126/science.272.5258.117 8600521

[B11] Hoppensteadt F. C. (2000). Analysis and Simulation of Chaotic Systems. New York, NY: Springer-Verlag.

[B12] Insperger T. (2015). On the approximation of delayed systems by taylor series expansion. J. Comput. Nonlinear Dyn. 10:024503 10.1115/1.4027180

[B13] Insperger T. Milton J. G. (2014). Sensory uncertainty and stick balancing at the fingertip. Biol. Cybern. 108, 85–101. 10.1007/s00422-013-0582-2 24463637

[B14] Insperger T. Milton J. G. Stepan G. (2015). Semidiscretization for time-delayed neural balance control. SIAM J. Appl. Dyn. Syst. 14, 1258–1277. 10.1137/140975632

[B15] Insperger T. Milton J. Stépán G. (2012). Acceleration feedback improves balancing against reflex delay. J. R. Soc. Interface 10:20120763. 10.1098/rsif.2012.0763 23173196 PMC3565692

[B16] Insperger T. Stepan G. (2010). On the dimension reduction of systems with feedback delay by act-and-wait control. IMA J. Math. Control Inform. 27, 457–473. 10.1093/imamci/dnq020

[B17] Kiyono K. Struzik Z. R. Aoyagi N. Sakata S. Hayano J. Yamamoto Y. (2004). Critical scale invariance in a healthy human heart rate. Phys. Rev. Lett. 93:178103. 10.1103/PhysRevLett.93.178103 15525130

[B18] Kiyono K. Struzik Z. R. Yamamoto Y. (2006) Criticality phase transition in stock-price fluctuations. Phys. Rev. Lett. 96:068701. 10.1103/physrevlett.96.068701 16606055

[B19] Kiyono K. Struzik Z. R. Yamamoto Y. (2007). Estimator of a non-Gaussian parameter in multiplicative log-normal models. Phys. Rev. E 76:041113. 10.1103/physreve.76.041113 17994942

[B20] Kloeden P. E. Platen E. (1992). Numerical Solution of Stochastic Differential Equations. Berlin; Heidelberg: Springer-Verlag.

[B21] Lakie M. Caplan N. Loram I. D. (2003). Human balancing of an inverted pendulum with a compliant linkage: neural control by anticipatory intermittent bias. J. Physiol. 551, 357–370. 10.1113/jphysiol.2002.036939 12832494 PMC2343154

[B22] Loram I. D. Gollee H. Lakie M. Gawthrop P. J. (2011). Human control of an inverted pendulum: is continuous control necessary? Is intermittent control effective? Is intermittent control physiological? J. Physiol. 589, 307–324. 10.1113/jphysiol.2010.194712 21098004 PMC3043535

[B23] Loram I. D. Lakie M. (2002). Human balancing of an inverted pendulum: position control by small, ballistic-like, throw and catch movements. J. Physiol. 540, 1111–1124. 10.1113/jphysiol.2001.013077 11986396 PMC2290269

[B24] Loram I. D. Maganaris C. N. Lakie M. (2005). Human postural sway results from frequent, ballistic bias impulses by soleus and gastrocnemius. J. Physiol. 564, 295–311. 10.1113/jphysiol.2004.076307 15661824 PMC1456055

[B25] Manshour P. Saberi S. Sahimi M. Peinke J. Pacheco A. F. Tabar M. R. R. (2009). Turbulencelike behavior of seismic time series. Phys. Rev. Lett. 102:014101. 10.1103/physrevlett.102.014101 19257196

[B26] Mehta B. Schaal S. (2002). Forward models in visuomotor control. J. Neurophysiol. 88, 942–953. 10.1152/jn.00804.2001 12163543

[B27] Milton J. G. Cabrera J. L. Ohira T. (2008). Unstable dynamical systems: delays, noise and control. EPL (Europhysics Letters). 83:48001 10.1209/0295-5075/83/48001

[B28] Milton J. G. Cabrera J. L. Ohira T. Tajima S. Tonosaki Y. Eurich C. W.. (2009). The time-delayed inverted pendulum: implications for human balance control. Chaos 19, 026110. 10.1063/1.3141429 19566270

[B29] Milton J. G. Small S. S. Solodkin A. (2004). On the road to automatic: dynamic aspects in the development of expertise. J. Clin. Neurophysiol. 21, 134–143. 10.1097/00004691-200405000-00002 15375344

[B30] Nomura T. Oshikawa S. Suzuki Y. Kiyono K. Morasso P. (2013). Modeling human postural sway using an intermittent control and hemodynamic perturbations. Math. Biosci. 245, 86–95. 10.1016/j.mbs.2013.02.002 23435118

[B31] Stepan G. (2009). Delay effects in the human sensory system during balancing. Philos. Trans. R. Soc. A 367, 1195–1212. 10.1098/rsta.2008.0278 19218159

[B32] Suzuki Y. Nomura T. Casadio M. Morasso P. (2012). Intermittent control with ankle, hip, and mixed strategies during quiet standing: a theoretical proposal based on a double inverted pendulum model. J. Theor. Biol. 310, 55–79. 10.1016/j.jtbi.2012.06.019 22732276

[B33] Yoshikawa N. Suzuki S. Kiyono K. Nomura T. (2015). Intermittent appearances of saddle-type hyperbolic dynamics during human stick balancing on a manually controlled cart. Conf. Proc. IEEE Eng. Med. Biol. Soc. 2015, 3500–3503. 10.1109/embc.2015.7319147 26737047

